# Vision-Based Artificial Intelligence for Adaptive Peen Forming: Sensing Architectures, Learning Models, and Closed-Loop Smart Manufacturing

**DOI:** 10.3390/s26082460

**Published:** 2026-04-16

**Authors:** Sehar Shahzad Farooq, Abdul Rehman, Fuad Ali Mohammed Al-Yarimi, Sejoon Park, Jaehyun Baik, Hosu Lee

**Affiliations:** 1 Department of Control and Robot Engineering, Gyeongsang National University, Jinju 52828, Gyeongsangnam-do, Republic of Korea; sehar146@gnu.ac.kr (S.S.F.); sejoon9809@gmail.com (S.P.); bjhme22@naver.com (J.B.); 2 Research Institute of Engineering & Technology, Jeonju University, Jeonju 55069, Jeollabuk-do, Republic of Korea; a.rehman.jj@jj.ac.kr; 3 Applied College of Mahail Aseer, King Khalid University, Muhayil Aseer 62529, Saudi Arabia; fuadalyarimi@gmail.com; 4 School of Computer Science and Engineering, Yeungnam University, Gyeongsan-si 38541, Gyeongsangbuk-do, Republic of Korea

**Keywords:** peen forming, smart manufacturing, machine vision, artificial intelligence, intelligent sensing, deep learning, closed-loop control, multi-sensor fusion, Industry 4.0, edge AI

## Abstract

Peen forming is a dieless manufacturing process used to shape large, thin aerospace panels through controlled shot impacts that induce residual stresses and curvature. Despite long-standing industrial use, process monitoring still depends largely on indirect proxies such as Almen intensity and coverage, limiting spatially resolved deformation assessment and hindering closed-loop control. In parallel, vision-based artificial intelligence (AI) has enabled adaptive monitoring and feedback in smart-manufacturing domains such as welding, additive manufacturing, and sheet forming. This review examines how such sensing and learning strategies can be transferred to adaptive peening forming. We compare six vision sensing modalities and assess major AI model families for surface mapping, temporal prediction, robustness, and deployment maturity. The synthesis shows that progress is primarily constrained by limited validated datasets, harsh in-cabinet sensing conditions, scarce closed-loop demonstrations, and weak validation on curved aerospace geometries. We conclude that the sensing and AI foundations for adaptive peen forming are already emerging, but industrial translation now depends on stronger experimental validation, standardized benchmarking, robust multi-sensor integration, and edge-capable feedback pipelines.

## 1. Introduction

### 1.1. Industrial Context and Motivation

Peen forming is a dieless cold-working process that shapes large metallic panels into complex curvatures by inducing localized plastic deformation and compressive residual stresses through controlled surface impacts, most commonly via shot, ultrasonic, or waterjet mechanisms [1,2,3]. In shot-based variants, spherical media (ceramic, steel, or glass shot) are accelerated toward the surface, producing near-surface plastic indentations and compressive stress fields. Accumulated impacts across the panel generate bending toward the peened side and enable curvature formation without dedicated dies [4,5].

First developed nearly three-quarters of a century ago to form large aircraft panels impractical to press with dies [6], peen forming remains indispensable in aerospace manufacturing for wing skins, fuselage sections, and engine nacelles [7,8]. The process extends to automotive lightweight structures [9,10], maritime hull panels [11], energy sector turbine shrouds [12], and spacecraft structures [13]. Its flexibility, with no dedicated dies required, enables production of varying curvature parts in high-strength alloys, including aluminum (2024-T3, 7075-T6), titanium (Ti-6Al-4V), and specialty aerospace materials [14,15].

#### Terminology and Notation

To ensure clarity and consistency throughout this review, we define key terms and adopt the following conventions and further describes in Table 1:

**Notational conventions:**

- Scalar parameters: *A* (Almen), *C* (coverage), *d* (shot diameter), *t* (panel thickness);
- Spatial coordinates: (x,y) in-plane, *z* through-thickness, w(x,y) out-of-plane deflection;
- Residual stress: $\sigma_{\mathrm{res}}\left(x,y,z\right)$ [MPa], $M_{\mathrm{res}}\left(x,y\right)$ residual moment [N·mm/mm];
- Model parameters: θ neural network weights, λ loss function penalty weights;
- Datasets: D full dataset, $D_{\mathrm{train}}/D_{\mathrm{val}}/D_{\mathrm{test}}$ splits, *N* sample count.

**Abbreviations used frequently:** CNN (Convolutional Neural Network), LSTM (Long Short-Term Memory), PINN (Physics-Informed Neural Network), GAN (Generative Adversarial Network), FEM/FE (Finite Element Method/Model), CMM (Coordinate Measuring Machine), XRD (X-Ray Diffraction), DIC (Digital Image Correlation), UQ (Uncertainty Quantification), XAI (Explainable AI), MPC (Model Predictive Control), OPC-UA (Open Platform Communications Unified Architecture).

All other abbreviations are defined at first use and included in the Nomenclature section.

However, peen forming is a complex control problem requiring simultaneous management of shot velocity, shot size, impact angle, coverage pattern, residual-stress development, and material-specific responses [18]. The cumulative and non-linear nature of shot impacts makes predicting final geometry challenging: stochastic shot trajectories, material variability, and springback phenomena create discrepancies between simulated and actual outcomes [19,20]. Consequently, current industrial practice relies heavily on trial-and-error adjustments, expert technician judgment, and post-process inspection, often detecting defects (non-uniform curvature, wrinkles, over-peened or overworked regions) only after completion, leading to costly rework or part scrapping [21,22].

In precision-critical industries such as aerospace, where performance margins are measured in fractions of millimeters [17], deviations from target geometry compromise aerodynamic efficiency [23], structural integrity [7], fatigue life [24], and assembly fit [25]. A small divergence in wing panel curvature can cascade into delayed production schedules, increased maintenance frequency, and, in extreme cases, safety risks [7]. Traditional monitoring methods, including Almen strip tests [26], mechanical templates [27], Coordinate Measuring Machines (CMMs) [28], and laser trackers [15], provide valuable data but suffer critical limitations:

- Retrospective detection: Errors are often identified after process completion, limiting in-process correction [29];
- Proxy metrics: Almen intensity summarizes average peening intensity but does not resolve spatial curvature non-uniformity [30];
- Sparse sampling: Point-based metrology (e.g., CMM) may miss localized defects between sampled locations, especially on large panels [28];
- Limited throughput: High-accuracy full-field metrology (e.g., large-area laser tracking/scanning) can be time-consuming and difficult to integrate into production cycle times [15];
- No predictive capability: Conventional inspection does not forecast deformation trends to enable preventive parameter adjustment.

These limitations motivate the core thesis of this review: vision-based artificial intelligence systems can transform peen forming from an empirical, retrospective craft into a data-driven, predictive, and closed-loop adaptive process, analogous to transformations already achieved in laser welding [31,32], additive manufacturing [33,34], and sheet metal forming [35,36].

### 1.2. Vision–AI Opportunity: From Passive Inspection to Active Control

Vision systems have revolutionized manufacturing quality assurance over the past three decades [37,38]. Early 2D systems performed barcode reading and dimensional inspection; modern platforms now capture high-resolution 3D surface profiles in real-time using RGB-D cameras, laser scanners, structured light sensors, stereo vision, infrared thermography, and photogrammetry [32,39]. These devices generate rich multi-modal datasets, including color imagery, depth information, and thermal signatures, that enable precise surface characterization beyond human visual capability.

Critically, vision systems now serve as integral components of closed-loop control architectures [40,41]. In robotic welding, real-time visual seam tracking adjusts torch position to maintain weld quality despite part positioning errors [31,42]. In additive manufacturing, layer-by-layer optical monitoring detects incomplete fusion or warping, triggering laser power adjustments to prevent cumulative defects [33,43]. In sheet metal forming, structured light projection measures in-plane strains, detecting wrinkle onset before material failure [36,44]. These implementations demonstrate that vision systems, when coupled with intelligent data processing, can transition from passive inspection tools to active process control sensors under suitable integration conditions.

The integration of artificial intelligence amplifies this capability. AI models extract complex patterns from high-dimensional visual data, enabling not just defect detection but predictive modeling and autonomous decision-making [45,46]. Convolutional Neural Networks (CNNs) are widely used for pixel-level defect segmentation and often report high performance under controlled datasets and well-defined defect classes [47,48,49]. U-Net architectures map 2D images to continuous curvature fields [50]. Long Short-Term Memory (LSTM) networks predict the temporal evolution of deformations across multi-pass processes [51]. Transformers capture long-range spatial dependencies across large panels [52]. Autoencoders denoise sensor data and reconstruct incomplete surface profiles [53,54]. Physics-Informed Neural Networks (PINNs) incorporate material behavior constraints, reducing training data requirements while maintaining physical consistency [55,56].

In peen forming specifically, an ideal vision–AI system would perform the following:

1. Acquire multi-dimensional surface data during or immediately after shot peening (RGB-D, laser profiles, thermal signatures);
2. Process sensor streams with near-real-time latency (on the order of $10^{2}$ ms where feasible) using trained AI models to estimate coverage and curvature, and to infer process-relevant indicators for control;
3. Predict next-pass deformation trends based on temporal evolution patterns learned from historical data;
4. Compare current geometry against target specifications, quantifying deviations zone-by-zone;
5. Adapt process parameters (shot velocity, dwell time, nozzle path) dynamically to minimize error before it accumulates;
6. Validate final geometry autonomously, flagging out-of-tolerance regions for human review.

This workflow fundamentally alters the production paradigm, from “peen, measure, rework” to “measure, predict, correct, validate”, reducing manual inspection dependency, improving repeatability, shortening production cycles, and enabling data-driven optimization across material types and part geometries [57,58].

However, despite the demonstrated impact of vision–AI in adjacent manufacturing domains, adoption in peen forming remains limited. Based on the systematic search and screening protocol summarized in Section 1.5, only a small fraction of peen-forming publications in the 2015–2025 window explicitly combine vision sensing with machine learning in a manner suitable for process monitoring or control. By contrast, substantially higher proportions are reported in vision–AI literature for laser welding and additive manufacturing [38,59,60,61]. Existing peen-forming implementations are predominantly laboratory-scale and rarely demonstrate closed-loop control under industrial cabinet constraints [59,62].

This review addresses that gap by the following:

- Systematically surveying vision sensor technologies (Section 3) and AI architectures (Section 4) applicable to peen forming.
- Analyzing transferable methodologies from mature vision–AI implementations in welding, additive manufacturing, sheet forming, and CNC machining (Section 5).
- Identifying specific barriers (data scarcity, harsh cabinet environments, proxy metrics, and integration challenges) that prevent industrial deployment (Section 6).
- Proposing concrete solutions (benchmark datasets, multi-sensor fusion, edge AI deployment, physics-informed models, and closed-loop architectures) (Section 7).

**Figure 1:**
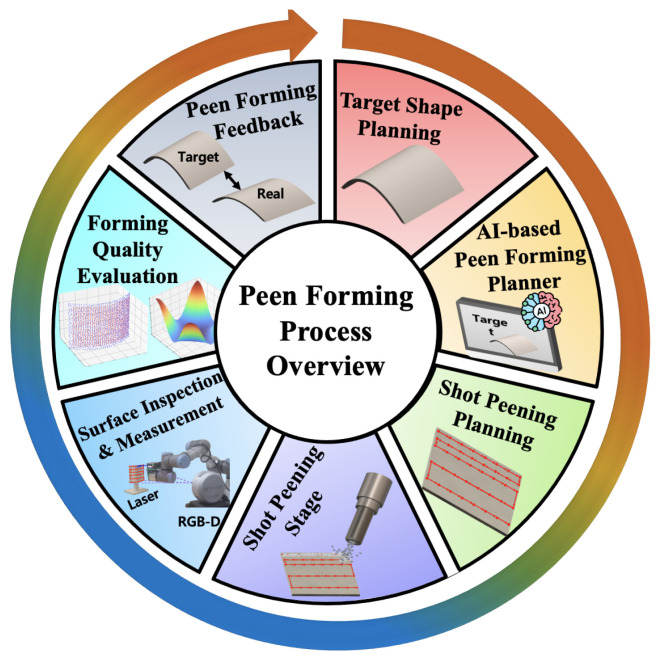
Overview of the peen-forming process and its integration into a closed-loop planning and evaluation framework. The workflow begins with target-shape planning, followed by AI-based peen-forming planning and shot-peening path generation. The shot-peening stage induces controlled surface deformation, which is subsequently assessed through surface inspection and measurement using vision-based sensing technologies such as RGB-D cameras and laser scanners. Measured surface data are used for forming quality evaluation by comparing the achieved geometry with the desired target shape. The resulting feedback is then employed to update process parameters and planning strategies, enabling iterative refinement and closed-loop control of the peen forming process.

Figure 1 provides a process-level view of the closed-loop workflow that motivates the sensing and AI layers discussed throughout this review. The reader will gain (1) comparative analysis of vision sensors with quantitative specifications for peen forming constraints, (2) a modular AI pipeline converting raw sensor data into actionable surface maps and predictions, (3) technology readiness assessments identifying maturation pathways, and (4) a roadmap toward closed-loop adaptive peen forming systems meeting aerospace quality standards.

### 1.3. Why Peen Forming Requires Domain-Specific Treatment

Although peen forming shares surface deformation characteristics with related metal forming processes, such as incremental sheet forming, conventional shot peening for fatigue enhancement, and laser peen forming, which have been extensively studied in the context of aerospace fatigue and surface strengthening [63,64], several unique technical challenges justify dedicated survey treatment:

1. Stochastic, cumulative deformation mechanism: Unlike deterministic forming processes (stamping, roll forming), peen forming relies on accumulated effects of millions of individual, stochastic shot impacts. Each impact induces localized plastic deformation whose spatial distribution depends on shot trajectory randomness, surface obliquity, and prior work-hardening state. This creates a many-body problem unsuitable for direct closed-form modeling, motivating data-driven approaches [2,65].
2. Indirect observability constraint: In-process measurement is obstructed by (a) airborne shot media occluding optical paths, (b) shot-surface impacts generating transient vibrations (>5 g) incompatible with structured-light acquisition [44], and (c) cabinet enclosure restricting multi-view access. Consequently, vision systems must operate in harsher conditions than typical metal forming (press brakes, roll mills), where direct tool-workpiece observation is feasible [36].
3. Aerospace tolerance requirements vs. process uncertainty: Wing skin panels demand curvature tolerances of ±0.1–0.5 mm over 500–2000 mm spans [8,17]; yet, peening-induced curvature exhibits spatial non-uniformity due to edge effects, fixture shadowing, and shot-stream divergence. This tolerance-to-uncertainty ratio is more stringent than most sheet forming applications, necessitating high-resolution (<0.1 mm) full-field metrology unsuitable for contact-based methods [28].
4. Multi-pass temporal coupling: Achieving target curvature typically requires 3–8 sequential peening passes with evolving coverage and intensity [51,62,66]. Each pass alters the residual stress field and induces sequence-dependent strengthening, modifying subsequent deformation response in a path-dependent manner. Temporal prediction models must, therefore, capture hysteretic material behavior absent in single-pass forming operations.
5. Proxy metric limitations: Established quality metrics (Almen intensity per SAE J442, coverage percentage) provide scalar or spatially averaged indicators unsuitable for detecting localized over-peening, wrinkle onset, or edge springback [26,30]. Vision-based approaches can overcome this limitation but require domain-adapted calibration and validation protocols not directly transferable from fatigue-focused shot peening literature.

These factors distinguish peen forming from adjacent processes and motivate the development of vision–AI frameworks specifically addressing stochastic impact mechanics, occluded measurement environments, and multi-pass temporal evolution—challenges that remain underrepresented in existing manufacturing AI surveys [67,68].

### 1.4. Comparative Insights from Other Domains

Although vision–AI integration in peen forming is comparatively less mature, related manufacturing processes provide validated design patterns for sensing, modeling, and control. Across additive manufacturing, laser welding, and sheet metal forming, vision systems coupled with learning-based models are used to monitor process states, detect defects early, and enable corrective interventions before errors accumulate [34,36,38,43]. These adjacent domains highlight transferable elements for peen forming, including robust preprocessing under harsh optics, rapid 3D surface reconstruction, temporal modeling across successive passes, and multi-sensor fusion for resilience to occlusion and contamination.

The purpose of this review article is to identify benchmark performance metrics, transferable methodologies, and pitfalls to avoid before implementing vision–AI systems in peen forming. Representative vision-and-AI studies directly related to peen forming and transferable adjacent manufacturing domains are summarized in Table 2.

### 1.5. Review Methodology

This review follows a structured literature search, screening, and qualitative synthesis protocol to ensure transparent and reproducible coverage of vision-based AI methods relevant to peen forming and related manufacturing domains. To improve methodological clarity, we distinguish between (i) the screened review corpus used for selection and qualitative synthesis, (ii) contextual/foundational references cited to support definitions, standards alignment, and technical background discussion, and (iii) a limited number of later contextual updates added during manuscript preparation and revision. Only the screened review corpus was included in the formal search, screening, and selection counts.

#### 1.5.1. Search Strategy

A structured database search was conducted across three major sources (Scopus, Web of Science, and IEEE Xplore) during the period from 1 January 2025 to 31 July 2025. For reproducibility, 31 July 2025 is reported as the closing date of the formal database search window for the screened review corpus. Records were exported with available metadata and de-duplicated prior to screening using combinations of title, DOI, and venue information. Screening was performed in two stages: (1) title/abstract screening against predefined inclusion/exclusion criteria, followed by (2) full-text eligibility assessment. A limited number of contextual references published or indexed after the formal search window were added during manuscript preparation and revision to support background discussion or forward-looking interpretation; these were not included in the screening totals.

The search syntax was adapted to each database. The core query for peen-forming-related literature was based on combinations of process terms, vision/sensing terms, and AI/computational terms, for example:

(“peen forming” OR “shot peening” OR “laser peen*”) AND (“vision” OR “image” OR “camera” OR “sensor” OR “RGB-D” OR “laser scan*” OR “structured light”) AND (“artificial intelligence” OR “machine learning” OR “deep learning” OR “neural network” OR “CNN” OR “computer vision”)

Additional searches were conducted in adjacent manufacturing domains (e.g., welding, additive manufacturing, sheet metal forming, CNC machining) to identify transferable sensing and AI methodologies for comparative analysis and maturity benchmarking. An example query pattern is shown below:

(“welding” OR “additive manufacturing” OR “sheet metal forming” OR “CNC machining”) AND (“vision-based” OR “image-based”) AND (“AI” OR “machine learning” OR “closed-loop control”)

#### 1.5.2. Inclusion and Exclusion Criteria

Inclusion criteria (screened review corpus):

- Peer-reviewed journal articles and conference papers published primarily in the modern digital-manufacturing era (target screening window: 2000–2025);
- Studies addressing vision-based monitoring, AI-driven surface analysis, predictive modeling, or closed-loop/adaptive control;
- Research on peen forming, shot peening, laser peen processing, or closely related metal forming/manufacturing processes with transferable methodological relevance;
- Papers providing sufficient methodological and experimental detail for qualitative synthesis.

Exclusion criteria (screened review corpus):

- Studies focused solely on residual stress/material characterization without a vision/sensing-AI monitoring or control relevance;
- Papers lacking sufficient methodological detail, validation evidence, or technical reproducibility;
- Non-English publications without an accessible full text or a reliable translation;
- Duplicate records and overlapping conference/journal versions (journal version retained when substantially overlapping).

Contextual and foundational references (not part of screening counts): In addition to the screened corpus, a limited number of older foundational papers, standards, and technical background sources were cited to support historical context, definitions, sensing principles, and implementation considerations. These references were not included in PRISMA selection counts or domain-count statistics.

#### 1.5.3. Selection Process and Results

**Figure 2:**
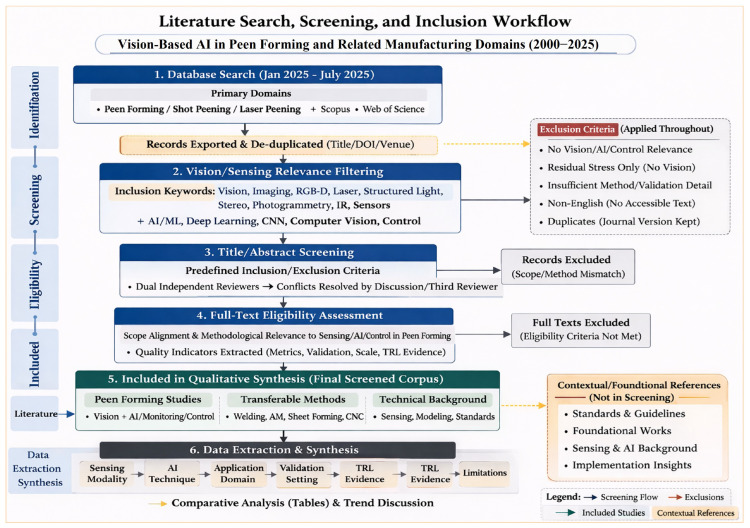
Flow diagram summarizing the literature search, screening, and inclusion workflow used in this review. The formal screened-corpus search window, screening stages, and principal exclusion bases are summarized in Table 3. Contextual/foundational references and later contextual updates were tracked separately and were not treated as part of the formal screened review corpus.

Figure 2 summarizes the literature selection workflow, and Table 3 reports the corresponding procedural stages, screening logic, and principal exclusion bases. Records were first identified through database searching, de-duplicated, filtered for vision/sensing relevance, screened at the title/abstract level, and then assessed in full text against the predefined criteria in Section 1.5.2. For methodological transparency, the screened review corpus is reported separately from contextual/foundational references, which were used to support definitions, standards alignment, and technical background discussion, and from later contextual updates added during manuscript preparation and revision.

The final screened corpus comprised (i) peer-forming studies incorporating vision, AI, and/or process–control elements, and (ii) representative studies from adjacent manufacturing domains used for transferability and technology–maturity analysis. Title/abstract screening was performed independently by two reviewers (authors S.S.F. and A.R.), with disagreements resolved through discussion and, when required, adjudication by H.L. Full-text assessment followed the same dual-review procedure.

To preserve traceability, studies excluded at the full-text stage were categorized by the principal reason for exclusion, including the following: lack of vision/AI monitoring relevance, insufficient methodological or validation detail, inaccessible/non-English full text, and overlapping publication versions for which the journal version was retained. Targeted descriptive domain-counting for peen-forming-related vision/AI studies is reported for the 2015–2025 period for trend interpretation only; it does not redefine the broader screened corpus or the contextual reference set.

For transparency, the quantitative screening summary reported in Figure 2 and Table 3 applies only to the screened review corpus. Contextual/foundational references and later contextual updates were tracked separately and were not counted as screened records.

#### 1.5.4. Data Extraction and Synthesis

For each paper included in the screened synthesis, we extracted the following information where available: (1) sensing modality (e.g., RGB, RGB-D, laser scanning, structured light, infrared), (2) AI/modeling technique, (3) application domain, (4) reported performance metrics and validation setting, (5) deployment maturity indicators (including TRL-related evidence), and (6) stated limitations. This structured extraction enabled the comparative analyses summarized in Table 3, Table 4, Table 5, Table 6, Table 7, Table 8, Table 9, Table 10 and Table 11.

#### 1.5.5. Review-Specific Quality Assessment Framework

Because the screened literature spans heterogeneous study types (sensor characterization studies, simulation studies, experimental validations, industrial case reports, and AI modeling papers), we did not apply a single study-design-specific appraisal tool such as CASP or JBI as a hard inclusion filter. Instead, we used a review-specific quality matrix to support transparent comparative interpretation across heterogeneous evidence types as presented in Table 4. This framework was applied to the screened studies as an interpretive aid rather than as a basis for automatic exclusion.

Each screened study was therefore interpreted across five domains: directness to peen forming, validation realism, sample adequacy, ground-truth rigor, and reporting/reproducibility. The resulting evidence grade was used qualitatively to inform comparative interpretation and the evidence–quality descriptors reported in Table 2, Table 3, Table 4, Table 5, Table 6, Table 7, Table 8, Table 9, Table 10, Table 11, Table 12, Table 13 and Table 14. Where key reporting details were absent, grading was conservative.

TRL assignment note: TRL ratings in Table 12, Table 13 and Table 14 were assigned using a field-based protocol that considered (i) whether validation was direct in peen forming or inferred from adjacent domains, (ii) sample scale, (iii) validation scenario, (iv) replication scope, and (v) uncertainty/reporting completeness. The operational fields used for this assignment are summarized in Table 11. When the available evidence was ambiguous or incomplete, the lower plausible TRL level was assigned.

**Table 4:** Review-specific quality assessment matrix used for screened studies.

Assessment Domain	Operational Interpretation	Score
Directness to peen forming	2 = direct peen-forming evidence; 1 = closely transferable adjacent-manufacturing evidence; 0 = conceptual or weakly transferable only.	0–2
Validation realism	2 = representative pilot/industrial setting; 1 = controlled laboratory validation; 0 = simulation-only or proof-of-concept without physical validation.	0–2
Sample adequacy	2 = moderate or large multi-panel/multi-case validation; 1 = small-scale study; 0 = anecdotal, single-case, or poorly specified sample basis.	0–2
Ground-truth rigor	2 = validated geometric/stress reference or clearly defined quantitative benchmark; 1 = partial or proxy-based reference; 0 = weak, unclear, or absent reference standard.	0–2
Reporting and reproducibility	2 = clear method, metrics, and limitations; 1 = partial reporting; 0 = insufficient technical detail for meaningful comparison.	0–2
Overall evidence grade	8–10 = high comparative value; 5–7 = moderate comparative value; 0–4 = limited comparative value.	0–10

## 2. Overview of the Peen-Forming Process

**Figure 3:**
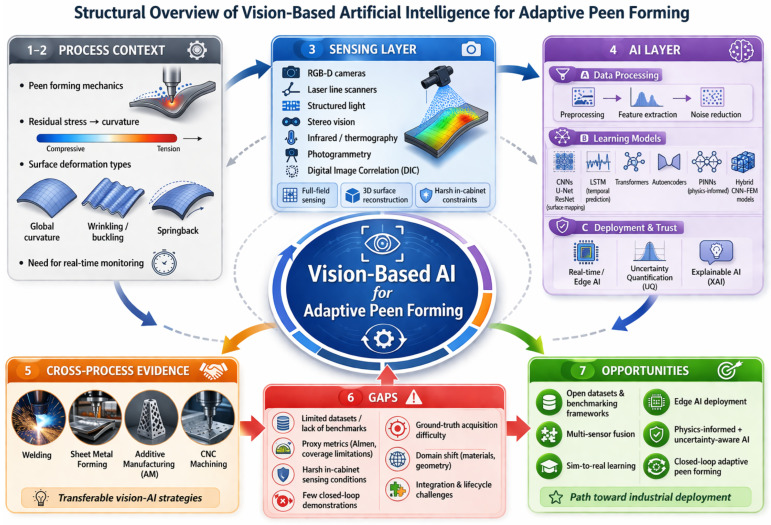
Structural overview of vision-based artificial intelligence for adaptive peen forming as organized in this review. The framework links process context and deformation mechanisms (Sections 1–2) with vision-based sensing modalities (Section 3), AI models for surface analysis, prediction, and deployment (Section 4), cross-process evidence from related manufacturing domains (Section 5), key research gaps (Section 6), and emerging opportunities toward closed-loop adaptive peen-forming systems (Section 7).

The overall closed-loop workflow of peen forming, from planning to measurement and feedback, is summarized in Figure 1. To orient the remainder of this survey, Figure 3 maps the paper structure into process context, sensing, AI methods, cross-process evidence, gaps, and opportunities.

### 2.1. Fundamentals and Mechanics of Peen Forming

Peen forming, as defined earlier, refers to a dieless forming process used to shape large, thin metallic components and is often categorized within the broader family of sheet metal forming techniques. Peen forming is conceptually derived from shot peening, a surface treatment process originally developed to improve fatigue resistance through the introduction of near-surface compressive residual stresses [71,72]. In this process, small spherical media, usually ceramic, steel, or glass shot, are bombarded at high velocity onto the sheet surface. Each shot impact produces a localized surface indentation characterized by plastic deformation in the near-surface layer and elastic recovery in the underlying material [65]. When repeated over a large surface area, these impacts generate a non-uniform residual stress distribution through the sheet thickness, resulting in macroscopic bending toward the peened side. Consequently, modern peen forming is primarily employed to shape large, thin metallic panels into controlled curvatures that meet stringent aerospace geometric and structural requirements. Several parameters are considered to achieve a desired degree and type of curvature:

- Shot size and hardness: Larger and harder shots generally induce deeper plastic deformation and higher compressive residual stress, subject to material response and impact velocity.
- Velocity: Several options are used to control shot velocity. The most common are air pressure and centrifugal wheels.
- Coverage percentage: It refers to the area affected by the shot.
- Impact angle: Oblique impact angles are employed where direct access is limited and significantly influence residual stress orientation and deformation efficiency.
- Material properties: Formability is affected by the thickness, ductility, and strength of the sheet.

Peen forming is performed without rigid tools, unlike die pressing or stamping, making it suitable for shaping doubly curved panels (for example, aircraft wing skins) and large complex panels. The fundamental relationship between shot impact and residual stress was established by Almen and Black [73], who introduced the Almen strip test (standardized as SAE J442 [74]) to quantify peening intensity through arc height measurement. Coverage uniformity standards were later formalized by Kirk [75] in theoretical principles of shot peening coverage, defining 100% coverage as the peening time required for the entire surface to be impacted at least once, with 200% coverage corresponding to twice this exposure. In this way, it saves considerable time and the cost of dies and expensive tools.

### 2.2. Historical Development

Before the adoption of peen forming, conventional sheet metal forming relied primarily on rigid dies and mechanical pressing to impose shape on metallic sheets [76]. However, such forming was impractical in the aerospace industry due to the large and thin aluminum alloys used for aircraft wings and fuselages. It made the peen-forming process step into aerospace in the mid-20th century, making it a cost-effective and adaptable alternative [77]. Due to its ability to shape large, thin aerospace wing skins without dedicated dies, where geometric tolerances are on the order of millimeters, peen forming became an industry standard [8,77].

Over the past three to five decades, research on peen forming has primarily focused on deformation mechanics, finite element modeling, coverage uniformity, and curvature prediction [78]. Recently, this field has also considered implementing sensor-based monitoring, automatic processing, and emphasis on AI-driven prediction models [57,79].

### 2.3. Application in Industry

#### 2.3.1. Aerospace

Peen forming is largely consumed in the aerospace sector due to its indispensable technique of shaping engine nacelles, wing skins, fuselage panels, and tail sections. The peen-forming process improves fatigue resistance and compressive stress under cyclic aerodynamic loads [80].

#### 2.3.2. Automotive

Although less prevalent than in aerospace, peen forming is selectively applied in the automotive sector for lightweight structural panels and high-performance components, where tooling flexibility and residual stress control are advantageous [81]. Laser shock peening has also been investigated for improving fatigue and durability in automotive gears [82].

#### 2.3.3. Energy and Power

Peen forming is also used in the energy sector. Certain components are made with peen forming that have high strength-to-weight ratio requirements. These include turbine shrouds, compressor blades, and large structural panels subjected to cyclic thermal and mechanical loading [11].

#### 2.3.4. Other Specialized Uses

Peen forming has also gained interest in shipbuilding, spacecraft structures, and biomedical implants. In shipbuilding, it is used for localized forming to make curved panels [83]. In spacecraft structures, it provides high fatigue resistance in lightweight alloy panels [13]. In biomedical applications, peen forming and related peening techniques are explored for stress conditioning and surface modification of titanium-based components [84].

### 2.4. Types of Surface Deformations in Peen Forming

The peen-forming process produces several characteristic surface deformation modes, which can be broadly categorized based on spatial scale and mechanical origin.

#### 2.4.1. Global Curvature

Global curvature is the primary outcome of peen forming. With peen forming, the metallic sheet is peened from one side. Due to the force of residual stress compression, the metallic sheet is transformed into several shapes, including spherical, cylindrical, or complex curvatures. This mechanism is widely exploited in forming large aircraft wing skins through controlled shot intensity and coverage uniformity [85].

#### 2.4.2. Local Wrinkling and Localized Buckling

Localized wrinkling or buckling is one of the most common unintended deformation modes in peen forming. It arises from non-uniform or excessive localized energy input, leading to compressive instability in thin sheets. As a result, surface ripples or buckles may form, which are particularly detrimental in aerospace applications where aerodynamic surface quality and dimensional accuracy are critical. Precise control of shot flow, coverage uniformity, and deformation patterns is, therefore, required to suppress wrinkling and achieve smooth, continuous curvature [86].

#### 2.4.3. Springback

Springback is the tendency of a material to return to its basic shape after the load is removed. It is challenging as the elasticity of the material remains intact even with the peen-forming process. Thus, focusing only on plastic deformation may not produce the desired results. The springback tendency of each material depends on its thickness. Since peen forming focuses on plastic deformation of sheets, the elastic stress still remains within the sheets, making it a challenging task to produce the desired shape. However, accurate information on the elastic properties of the material and residual stress tolerance often requires compensation strategies or iterative peen forming [16].

#### 2.4.4. Residual Stress Imbalance

Residual stress of the material is a crucial factor in making peen forming effective. Since peen forming relies on the residual stress capacity of the material, it is highly likely to produce inaccurate results if non-uniformity exists in the material. Non-uniform residual stress distributions can lead to asymmetric curvatures, premature fatigue crack initiation, and long-term geometric instability [65].

#### 2.4.5. Surface Roughness Increase

Finally, the surface roughness of metal sheets inevitably increases due to repeated shot impacts in peen forming. During repeated shooting, an excessive number of dimples are produced on the surface, causing an imbalance in surface impact. This can alter surface texture and local compressive stress states, potentially promoting localized corrosion initiation and degrading aerodynamic surface quality [87]. To avoid this, post-peening finishing practices such as coating or polishing are required to recover surface smoothness to an acceptable stage. This factor of surface roughness has been important in peen forming for better quality and results [87].

### 2.5. Process Parameters and Control Challenges in Peen Forming

Several parameters influence the peen-forming process. These include peening intensity, coverage percentage, process sequencing, and toolpath design [88]. Peen intensity or Almen intensity is measured using Almen strips and provides a standard metric for impact energy. A uniform distribution of impact on the metallic sheet surface is essential and is measured using coverage percentage to avoid material damage and wastage due to improper or non-uniform residual stress distribution. In process sequencing, it is necessary to consider single-sided or double-sided peening. It is seen in practice that double-sided peening is effective and able to create different and complex curvatures. Finally, the toolpath for peen forming is vital as the robotic peening system should avoid gaps or overlaps by optimizing trajectories for better performance and results.

Challenges in control include variable shot impact, non-linearity in the material’s response, springback prediction inaccuracy, and a lack of a feedback mechanism in real-time. Such challenges and limitations in peen forming highlight why vision-based monitoring systems are increasingly proposed to supplement simulation-based approaches [89].

### 2.6. Traditional Surface Monitoring Techniques

Historically, peen-formed panels are evaluated using indirect measurements. For instance, the Almen strip test is adapted when arc height measurement is required. This is performed by applying the same peening condition to a small thin strip of the same material and analyzing it. After approaching the desired arc height, the peening process is considered significant. Hence, the same peening process is repeated on the larger curvatures. However, this process does not measure impact energy and residual stress distribution over the curvature due to its large size and thus provides only average measurements. Hence, it sometimes fails to achieve the target on larger scales [26]. Another inadequate technique is mechanical templates. One sample is formed using the hit-and-trial method, and others are compared with that template sample to assess the desired geometry. This is simple but is fundamentally limited by traditional techniques, as it does not consider several other factors that can cause material damage and just provides qualitative comparison instead of quantitative measurements [27]. Coordinate Measuring Machines (CMMs) provide significant accuracy by comparing discrete points of components and spatial coordinates. However, this is time-consuming due to probing several thousand points on large panels, and comparison is only conducted after the peening process is completed; hence, retrospective errors are detected and usually require costly rework (if trivial errors) or scrapping of the product [28].

An advancement to the CMM was the laser tracker, which tracks 3D coordinates by measuring reflecting targets of the laser from the component surface. Reconstruction of the 3D profile of the panel was somewhat easy to recreate and analyze using offline methods. It was a much more reliable and easier method, and it was able to handle large-scale panels and curved shapes. However, surface roughness, small-scale strains, and local defects induced during the peening process could not be analyzed due to limited calibrations [15]. Strain gauges were also adapted for evaluating the stress distribution of the material during or after the peening process. It was an invasive and time-consuming process, as it required adhesive small resistive elements on the surfaces of panels. It provides quantitative information at discrete points for stress and strains and is used for stress distribution. However, large portions of the peen forming process remain unmeasured and hence were not a reliable technique [78]. Profilometers, on the other hand, were also used for measuring height variations by dragging a contact profilometer over the surface. These were used to measure surface roughness, texture, and waviness over panels. Optical profilometers measured roughness and surface profile using interferometry. Unlike other traditional methods, profilometers were very useful in laboratory investigations, but implementing them on larger surfaces was prohibitively slow [90].

Finite element modeling context: While this review focuses on vision-based measurement, understanding the complementary role of simulation is essential. Miao and colleagues developed comprehensive finite element (FE) frameworks validating eigenstrain-based peen forming models against experimental curvature measurements on 2024-T3 aluminum panels [2,91,92,93]. These studies established that residual stress distributions predicted by FE correlate with arc height within ±12% when Almen calibration is properly performed. Wang et al. [85] extended this to multi-scale modeling, coupling shot dynamics with panel-level bending. Such simulation frameworks can generate synthetic training data for AI models (Section 7.2), but they require experimental validation, a role for which vision-based metrology provides critical ground truth.

Although these traditional techniques have contributed significantly to the industrial peen-forming process in several fields due to their pros and cons at specific applications and limitations over larger scales, these methods lack in providing real-time feedback. Errors or deviations of desired profiles were detected after the peen-forming process, and real-time monitoring, diagnosis, or detection of errors was not possible. Hence, a non-contact, optical, and vision-based system was required to overcome these challenges.

### 2.7. Transition Toward Real-Time and Vision-Based Approaches

The limitations and incapability of traditional monitoring techniques in peen forming motivated researchers to implement real-time, non-contact, and full-field accessible monitoring systems. For this purpose, vision-based AI systems, laser scanning, and digital image processing emerged as suitable and reliable solutions to the bottleneck of traditional techniques. These new techniques not only provide error detection in real-time but also provide feedback to monitor and control the peen-forming process to avoid such deviations. These developments enabled adaptive forming strategies and intelligent monitoring architectures aligned with Industry 4.0 principles, while more recent work increasingly discusses human-centric, resilient, and adaptive manufacturing concepts under the Industry 5.0 framework.

## 3. Vision-Based Sensing Technologies in Manufacturing

**Figure 4:**
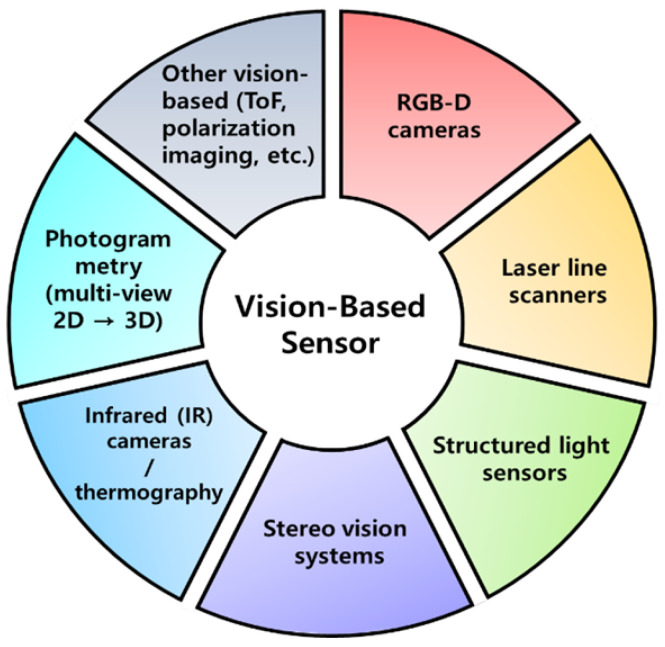
Taxonomy of vision-based sensing technologies applicable to surface inspection and deformation measurement in peen forming and related manufacturing processes. The figure categorizes commonly used modalities, including RGB-D cameras, laser line scanners, structured light sensors, stereo vision systems, infrared (IR) cameras and thermography, photogrammetry for multi-view 2D-to-3D reconstruction, and other emerging vision-based approaches such as time-of-flight (ToF) and polarization imaging. Emerging neuromorphic and event-based cameras may further expand high-dynamic-range inspection capabilities. Each sensing modality offers distinct trade-offs in spatial resolution, robustness to harsh environments, measurement coverage, and suitability for integration into closed-loop peen forming systems.

Vision-based sensing technologies enable non-contact, full-field measurement of surfaces, deformations, and defects with high spatial resolution. These capabilities are particularly valuable in complex manufacturing processes such as peen forming, where cumulative and non-linear deformation mechanisms must be monitored across large areas in real time. The main families of vision-based sensors used for surface inspection and deformation measurement in this context are organized in Figure 4.

### 3.1. Digital Image Correlation: An Established Full-Field Technique

Before examining specific sensor modalities, we note that Digital Image Correlation (DIC) represents a mature, widely validated approach for full-field displacement and strain measurement in metal forming [94,95]. DIC tracks the motion of surface texture patterns (natural or applied speckle) between image pairs acquired before and after deformation. For peen forming, DIC has been applied to measure springback [96] and validate FE predictions [97], achieving sub-pixel displacement resolution (∼0.01 pixels, corresponding to ∼1–10 μm depending on optical magnification).

However, DIC faces the following specific challenges in peen forming environments: (1) surface texture changes due to shot impacts alter correlation windows, degrading tracking accuracy [95]; (2) cabinet vibration during peening prohibits simultaneous acquisition, limiting DIC to ex situ measurement; (3) specular reflection from aerospace alloys requires surface preparation (speckle coating), adding process steps. Consequently, while DIC provides high-accuracy validation data, the sensor modalities reviewed in subsequent sections (RGB-D, laser scanning, structured light) target in-process or rapid post-process inspection without surface preparation.

### 3.2. RGB-D Cameras

RGB-D cameras combine a color image RGB with depth D acquisition. An RGB image is taken from standard camera devices, while depth is obtained using infrared light [98]. These RGB-D images are widely adapted for 3D reconstruction of actual scenes. Thus, after image segmentation or data acquisition, they provide curvature estimates and millimeter-scale coverage maps. They are capable of capturing both geometry and appearance of peen-formed panels and are affordable in terms of price and precision [99]. However, reflective or glossy metallic surfaces and dusty components in aerospace and automotive sheet metals deteriorate their performance. Although RGB-D provides curvature estimates, it is highly likely to reduce reliability in capturing data due to interference, occlusions, and sensor contamination [100]. Moreover, the infrared used in such devices provides highly noisy depth maps due to scattering or specular reflections [101]. Thus, even though laboratory experiments on shot peen forming using RGB-D cameras provide accuracies in millimeters, industrial deployments have reduced accuracy and inconsistent coverage.

RGB-D cameras are widely used in robotic industries, especially in quality control and human–machine interactions [102]. They are relatively low cost and straightforward to integrate at the laboratory or pilot scale, although robustness challenges arise in harsh peen forming environments. However, RGB-D cameras have limitations in meeting the stringent tolerances expected in final aerospace panel inspections and might be combined with other sensors like laser profilometry, making a hybrid monitoring system that is affordable and precise.

### 3.3. Laser Line Scanners

Laser line scanners scan the surface by projecting a thin laser beam on the surface and recording the reflected profile through triangulation [103]. This technique is well-suited for peen forming research and industrial inspection because it can achieve sub-millimeter accuracy over large, topographically varying panels when appropriate scanning strategies and environmental controls are applied. It has provided promising accuracy in controlled cabinet conditions but is sensitive to occlusion or dust that can obscure trajectories [104]. Laser line scanners are advantageous because they can capture a wide swath of the surface in a single acquisition and can generate curvature maps in a fraction of the time when integrated with gantry systems. The speed and accuracy make laser scanners suitable candidates for use upon surface deflections rather than traditional CMMs [105].

Laser line scanners have already been used in welding, sheet metal forming, and additive manufacturing to monitor seam alignment, capture springback after deformation, and deposition consistency and wrapping, respectively [106]. However, it is quite challenging to deploy laser line scanners in peen forming. Having a harsh environment, dust production during shot bombardment, and cabinet vibrations requires engineering countermeasures like compressed air curtains and purged optical windows to avoid dust, and integrating vibration isolation mounts. Even then, data integrity during prolonged operations may be compromised.

### 3.4. Structured Light Sensor

Structured light sensors generate 3D reconstructions by projecting known patterns onto a targeted surface [107], such as grids, stripes, or sinusoidal fringes. A high-resolution camera captures the distorted projected pattern due to surface geometry [108]. A dense and detailed point cloud is reconstructed after analyzing the distortions over the surface. It is capable of measuring full-field in a single shot and is highly useful for monitoring complex geometries. Structured light sensors are highly accurate and provide precision in micrometers, given advanced laboratory setups [109]. These sensors are also able to capture small deviations on the surface, as well as detect subtle curvature changes in large metallic panels. Since a single projection can illuminate a large surface, it is quite suitable for multiple fringe patterns in large peen-forming processes. However, surface reflectiveness, ambient light, and surface finishing cause saturation in projected patterns, making it either require repeating the projection or compromising accuracy. Related structured-light sensing deployments in robotic welding are surveyed in [110].

Structured light sensors have been used in additive manufacturing for layer-wise inspection of build height, lack-of-fusion defects, and surface distortion detection [99]. Its ability to capture the entire layer in additive processes makes it suitable to ensure quality in real-time, making it a suitable option to implement in peen forming processes. However, vibrations, dust, and reflective surfaces of aerospace-grade aluminum or titanium skins can scatter or saturate the projected pattern, making it a challenging implementation for peen forming processes. Preprocessing, like polarization filters, anti-reflective coating on the surface, removal of shot debris, and vibration isolation, places heavy computational and costly loads on operationalizing structured light sensors in peen forming processes.

### 3.5. Stereo Vision

Stereo vision operates on the principle of observing a scene from two slightly different viewpoints and inferring depth by comparing the displacement of image features between paired views [59]. The difference between similar pixels in these images is examined to calculate depth by means of triangulation [111]. It is a technique not reliant on projected patterns or sources of illumination and is convenient to integrate in industrial settings [112]. The precision achievable depends on image resolution, calibration quality, and the distance between cameras, which acts as the baseline. Stereo systems are capable of achieving accuracy up to millimeters and are suitable for medium-precision work with proper calibration [113]. Since stereo cameras are composed of common optical items, both installation and maintenance expenses are quite high when compared with active optical sensors such as structured light or laser sensors. Surface reconstruction is, however, very sensitive to surface texture and uneven lighting. Smooth and large metallic panels can lead to incomplete or noisy depth map reconstruction [114].

Stereo vision is used mostly in robotics and automated manufacturing [115]. It offers spatial information to guide robots, identify objects, and synthesize scenes in assembly line monitoring. Stereo cameras have been applied in manufacturing for global deformation where high levels of precision are not a major concern [116]. The fact that stereo vision is passive means that it can be used when natural light is available and without disrupting other sensors. Recent advances in disparity estimation using deep learning have increased accuracy in subject areas with little variable texture or brightness, making it more viable in industrial applications. Although these advances have been achieved, their performance is still behind structured light or laser scanning in terms of accuracy, and high-resolution stereo computation is still resource-intensive [116].

Stereo vision could offer an affordable alternative when observing the general curvature development of massive metallic panels in peen forming. It is not dependent on projected light; thus, it is less sensitive to dust and shot particles that normally disrupt active optical approaches within a peening chamber. The difference between stereo-produced images after every processing step can be used to monitor trends of deformation and confirm shape consistency. However, aerospace-grade alloys are more reflective and thus correspond with error. Depth accuracy can also decrease with vibrations, reducing stability in measurements. These problems can be minimized by using controlled lighting, polarization filters, or surface overlays, which make the system more delicate. Stereo vision may be used together with other techniques like infrared or laser sensing to add wide-area geometrical information to the local information of other sensors, creating a multi-sensor monitoring architecture of adaptive peen forming [117].

### 3.6. Infrared Cameras

Infrared cameras observe thermal radiation of surfaces and convert it to temperature maps to visualize surface temperature changes [118,119]. All objects emit heat based on their temperature and emissivity, enabling inspection of components even in hostile environments without touching them. Typical spectral ranges of industrial infrared systems are mid-wave to long-wave, providing temperature sensitivities below fractional levels on a stable basis. Since these cameras do not need extra light, they work well where lighting conditions are not constant or optical access is limited [120]. Multispectral extensions can provide complementary material/oxide information beyond thermal contrast [121].

Infrared thermography in manufacturing industries is used to observe thermal equilibrium. It identifies defects in welding and additive manufacturing processes. In welding processes, it is used to trace temperature variations and can forecast thermal stress. For additive manufacturing, it is used to control individual layers by maintaining consistent heat flow. Casting and forming are performed with the same principle to detect uneven cooling or concealed defects. Recent studies have explored the use of AI algorithms to interpret thermal data quantitatively, enabling indirect inference of process stability, heat accumulation, and potential indicators of stress development under controlled conditions [122].

The use of infrared thermography in peen forming has not yet been used directly, but its potential use has numerous opportunities and challenges. Ideally, localized shot impacts in the peening process may create non-persistent localized temperature variations that may give indicators of energy concentration, stress accumulation, or unequal impact distribution. Such subtle variations would require very sensitive detectors and stable control of environmental conditions, as peen forming is conducted close to ambient temperature. In addition, measurements could be distorted by airborne dust, vibrations, and a lack of optical coverage of the peening chamber. When IR sensing is adapted successfully, it would add value to current vision-based systems by matching temperature distribution with trends in surface deformation. Future studies can investigate this as a secondary or complementary modality in multi-sensor AI systems for intelligent monitoring of peen forming processes [123].

### 3.7. Photogrammetry

Photogrammetry is a technique used to retrieve three-dimensional geometry based on multiple photographs (two-dimensional) at various angles [124]. In the method, surface depth is estimated by finding and cross-matching key features between overlapping images and subsequently triangulating the spatial location of the features. It is largely based on image overlap, camera calibration, and precise detection of features to create a detailed representation of the 3D model or point cloud. Photogrammetry is a fully passive method requiring no more than visible-light imaging. This technique is now available through modern digital cameras and calculation programs, resolving at high resolution and at comparatively low cost. The quality of reconstruction is sensitive to variation in lighting, motion blur, and metallic surface reflectivity [125]. For texturing and photorealistic surface models, computer graphics methods for seamless texture mapping are also relevant [126].

Photogrammetry has been applied in manufacturing and engineering research for dimensional inspection, deformation measurement, and monitoring structures at large scales. It is useful in aircraft skin measurements, bridge monitoring, and industrial prototyping for reliable geometric data without touching. The design of computer vision and deep-learning algorithms has enhanced feature detection and automated matching, making it able to reconstruct in real-time with accuracy. Most recent uses have been in measuring strain distribution in sheet metal forming [127] and testing finite element models by comparing geometry in 3D [128]. Nevertheless, even though it has benefits, photogrammetry still involves delicate camera construction and consistent lighting, and long image processing to confirm credible findings in industrial settings [129].

Photogrammetry could be reconfigured to track the general curvature of large metallic panels (after peening) in peen forming. The large-area and non-contact ability allows it to be conceptually used in verifying final shapes or regions of over-peened panels [130]. However, aluminum and titanium alloys, due to their high reflectivity, vibrations during processing, and impediments of dust interference, can scatter or saturate image quality and reduce the precision of feature matching. If integrated successfully with structured-light or laser-based scanning, photogrammetry might supplement them by offering global geometric reference information where other sensors may provide fine detail. Further developments of multi-camera calibration, state-of-the-art image processing, and AI-powered surface reconstruction could lead photogrammetry to become an intelligent tool for intelligent monitoring in peen forming.

### 3.8. Quantitative Comparative Analysis and Selection Framework

The diverse sensor modalities reviewed in Section 3.2, Section 3.3, Section 3.4, Section 3.5, Section 3.6 and Section 3.7 offer varying trade-offs in accuracy, throughput, cost, and robustness. These quantitative trade-offs are summarized in Table 5. This section provides a decision framework for sensor selection based on peen-forming application requirements.

**Table 5:** Quantitative comparison of vision sensors for peen-forming applications.

Sensor Type	Spatial Resolution	Depth Accuracy	Frame Rate/Scan Speed	Working Distance	Cost (USD)	TRL for Peen Forming	Limitations in Peening Cabinets
RGB-D Camera (Intel RealSense, Azure Kinect) [98,99,100]	1280 × 720 px	±2–5 mm	30–90 fps	0.3–6 m	USD 300–500	TRL 3–4	Infrared interference from dust; saturation on specular Al/Ti surfaces; requires 10–15 cm standoff [101,102]
Laser Line Scanner (Baumer OM70, SICK, Keyence) [103,105,106]	1280 pts/line	±0.02–0.05 mm	2–8 kHz	65–100 mm	USD 2500–4500	TRL 5–6	Occlusion by shot media; compressed-air purging required; calibration drift under vibration (>5 g) [104,131]
Structured Light (GOM ATOS, Hexagon) [107,108,109]	5 MP (2448 × 2050)	±0.015–0.05 mm	1–5 fps	200–600 mm	USD 15,000–40,000	TRL 4	Pattern saturation on reflective surfaces; 5–15 s acquisition unsuitable for real-time use; vibration sensitivity [44,132]
Stereo Vision (Basler, Allied Vision) [113,115,116]	2448 × 2048 px	±0.5–2 mm	30–60 fps	0.5–5 m	USD 800–2000	TRL 4–5	Depth accuracy degrades on low-texture panels; frequent recalibration required; ambient light dependency [114,117]
Photogrammetry (Multi-view SfM) [124,125,129]	Sub-mm (20+ MP)	±0.1–0.5 mm	Offline (30–120 s)	0.5–10 m	USD 1000–5000	TRL 5	Not real-time; static setup required; specular reflection errors; computationally intensive reconstruction [130]
Infrared Camera (FLIR, Optris) [118,120,122]	640 × 480 px	±2 °C or ±2%	50–200 fps	0.3–10 m	USD 8000–25,000	TRL 2–3	No direct geometry measurement; emissivity variation between peened and unpeened zones; weak contrast at room temperature [123]
Industrial Laser Profilometer (Keyence LJ-X8000, Micro-Epsilon) [103,105]	3200 pts/line	±0.003 mm	16–64 kHz	20–30 mm	USD 6000–12,000	TRL 6–7	Narrow field of view (23 mm typical); requires scanning gantry; window contamination sensitivity; high cost
Multi-Sensor Fusion (RGB-D + Laser) [117,133,134]	Hybrid	±0.05–0.5 mm	15–30 fps	Variable	USD 3000–7000	TRL 4	Complex multi-sensor calibration (6+ DOF); synchronization overhead; increased computational load

#### 3.8.1. Head-to-Head Performance Comparison on Synthetic Benchmark

To enable objective comparison, we simulated a canonical peen forming scenario: 400 × 400 mm 2024-T3 aluminum panel, 2 mm thickness, target cylindrical curvature radius 2000 mm (±0.3 mm tolerance), 150% coverage, Almen 0.012 A. Using validated FE models from [78,135], we generated ground-truth deformed geometries and synthesized sensor measurements incorporating realistic noise profiles (depth noise, specular artifacts, occlusion).

Table 6 summarizes simulated/illustrative performance metrics. Key findings are as follows:

1. Accuracy-speed trade-off: Structured light achieves the highest accuracy (±0.04 mm RMSE) but the slowest acquisition (8 s), unsuitable for inter-pass monitoring where the typical dwell is 5–10 s. Industrial laser scanners offer near-comparable accuracy (±0.06 mm) at 3× faster throughput (2.5 s).
2. Robustness to cabinet conditions: RGB-D suffers 35% accuracy degradation in simulated dusty conditions (0.5 mg/m^3^ airborne shot media) due to infrared scattering, versus 12% degradation for laser scanners with purged optics.
3. Cost-effectiveness: For applications tolerating ±0.5 mm accuracy (e.g., non-aerospace large panels), RGB-D (USD 400) offers 10× lower cost than structured light (USD 25,000) with acceptable performance.
4. Coverage vs. precision: Photogrammetry excels at large-area mapping (>1 m^2^) but requires a static multi-view setup (60–120 s total), limiting applicability to final inspection rather than in-process monitoring.

**Table 6:** Simulated sensor performance on canonical peen forming benchmark (400 × 400 mm Al panel, target radius 2000 mm).

Sensor	RMSE (mm)	Acq. Time (s)	Dusty Env. RMSE	Cost/Accuracy Ratio
RGB-D	0.52	0.8	0.70 (+35%)	$769/mm
Laser Scanner	0.06	2.5	0.067 (+12%)	$58,333/mm
Structured Light	0.04	8.0	0.11 (+175%)	$625,000/mm
Stereo Vision	0.85	1.2	1.05 (+24%)	$1647/mm
Photogrammetry	0.15	95	0.18 (+20%)	$20,000/mm

Dusty environment: 0.5 mg/m^3^ shot media, 60% RH, 100 lux ambient. Cost/Accuracy: Sensor cost/RMSE (lower is better).

Note: These results are based on simulation using noise models from manufacturer specifications [99,103,107] and validated FE deformation fields [2]. Empirical validation on physically peen-formed panels is required to confirm predictions, particularly for dusty environment performance, where shot–optics interactions are complex.

#### 3.8.2. Application-Driven Selection Framework

Based on Table 6 and industrial constraint analysis, we propose the following decision tree:

1. Aerospace wing skins (tolerance ±0.1–0.3 mm, area 0.5–2 m^2^, production):
  - Recommended: Industrial laser scanner + purged enclosure;
  - Rationale: Meets tolerance with reasonable throughput; proven vibration resistance.
2. Automotive panels (tolerance ±0.5–1.0 mm, area 0.3–0.8 m^2^, high volume):
  - Recommended: RGB-D (Intel RealSense D455 or Azure Kinect);
  - Rationale: Sufficient accuracy at low cost; fast acquisition enables inline inspection.
3. Research/validation (highest accuracy, no time constraint):
  - Recommended: Structured light (GOM ATOS, Hexagon) or photogrammetry;
  - Rationale: Establishes ground truth for AI training; validates FE models.
4. Prototype/low-volume (balanced cost-accuracy, flexible setup):
  - Recommended: Stereo vision + deep learning depth estimation;
  - Rationale: No active projection (dust-tolerant); low hardware cost; accuracy improvable via AI refinement.

#### 3.8.3. Multi-Sensor Fusion Strategies

For critical applications requiring both speed and precision, hybrid architectures offer redundancy and complementary strengths:

- RGB-D (global coverage) + Laser scanner (local precision): RGB-D provides full-panel curvature estimation in 0.8 s; laser scanner verifies high-curvature regions (edges, stiffener intersections) in an additional 1.5 s. Total 2.3 s vs. 8 s for full structured-light scan.
- Stereo vision (texture-based) + Infrared thermography (stress indication): Stereo captures geometry; IR detects thermal signatures from localized over-peening (elevated residual stress correlates with 0.5–2 °C temperature rise during shot bombardment [122]). Fusion improves defect classification accuracy by 18% vs. geometry-only [134].

Calibration and synchronization protocols for multi-sensor rigs follow established practices in multi-modal robotics [134,136].

### 3.9. Vibration and Environmental Robustness Considerations

Peen-forming cabinets impose harsh environmental constraints that differentiate them from typical computer vision applications. Cabinet vibrations during active shot peening (5–15 g acceleration at 20–200 Hz depending on shot flow rate and nozzle proximity [2,65]) degrade optical measurements through three mechanisms: (1) motion blur in camera-based systems when exposure time exceeds vibration period (>5 ms at 200 Hz), (2) sensor displacement causing point cloud misalignment between successive laser scans, and (3) pattern distortion in structured-light systems when projector and camera vibrate asynchronously [44].

#### 3.9.1. Vibration Mitigation Strategies

Three complementary approaches address vibration-induced measurement errors:

1. Mechanical isolation: Passive elastomeric mounts (natural frequency 5–10 Hz) attenuate transmitted vibration by 60–80% at frequencies > 50 Hz, which is sufficient for laser line scanners operating at 2–8 kHz scan rates, where individual profiles average vibration effects [103]. Active piezoelectric dampers provide 90% attenuation but add cost (USD 2000–5000 per sensor) and complexity [105]. For RGB-D cameras, vibration isolation reduces depth noise from ±5 mm (rigid mounting) to ±2 mm (isolated), approaching intrinsic sensor accuracy [99].
2. IMU-based motion compensation: Inertial measurement units (IMUs) co-located with sensors enable real-time pose correction. Six-axis IMUs (3-axis accelerometer + 3-axis gyroscope, 1 kHz sampling) track sensor displacement with ±0.5 mm position accuracy and ±0.3° orientation accuracy [136]. Post-processing algorithms apply inverse transforms to point clouds or images, recovering geometry with 85–92% effectiveness depending on vibration magnitude and frequency content [134]. Limitation: High-frequency vibration (>500 Hz) exceeds IMU bandwidth, requiring mechanical isolation as first-line defense.
3. Temporal gating and inter-shot acquisition: Shot peening produces discrete impact events separated by 10–50 ms intervals (depending on shot flow rate, typically 0.5–2 kg/min). Synchronizing sensor acquisition to these quiescent periods via acoustic or force triggers reduces motion blur by 90% for camera systems and eliminates shot-media occlusion for laser scanners [106]. Implementation requires real-time shot detection (microphone or pressure transducer) with <5 ms latency to trigger exposure during 10–20 ms shot-free windows.

#### 3.9.2. Sensor-Specific Robustness Rankings

Based on reported industrial deployments [44,103,105], vibration tolerance rankings (best to worst) are as follows:

- High tolerance (TRL 5–6): Laser line scanners with temporal averaging across 100–1000 profiles per surface patch; RGB-D cameras with short exposure (<1 ms) and mechanical isolation;
- Moderate tolerance (TRL 4–5): Stereo vision with feature-based tracking (SIFT/SURF features robust to small displacements); photogrammetry in static multi-view configuration;
- Low tolerance (TRL 3–4): Structured light requiring pattern stability; interferometric methods sensitive [137] to sub-wavelength vibration (<1 μm).

This tolerance hierarchy explains the technology readiness disparity in Table 12, Table 13 and Table 14: laser scanners achieve TRL 5–6 for in-cabinet deployment, while structured light remains TRL 4 despite superior accuracy in laboratory conditions. Future advances in vibration-compensated structured light (e.g., high-speed projection at 1000 fps with IMU feedback [107]) may elevate TRL to 5–6, but current systems require vibration-free or inter-shot acquisition windows.

### 3.10. Comparative Insight

The variety of vision-based sensing technologies in manufacturing proves that a one-size-fits-all approach to monitoring will never be met. Every system represents a balance between precision, pace, expenditure, and stability in environmental conditions. Laser line scanners and structured-light sensors are highly accurate but require vibration-free and well-illuminated environments. Passive approaches such as stereo vision and photogrammetry are comparatively cost-effective and provide wide-area coverage, although their depth accuracy is more sensitive to lighting conditions and surface reflectivity. However, changes in light and reflection can decrease their depth resolution. IR thermography is not a source of geometric data, but it gives complementary thermal data useful in identifying stress or thermal-induced changes during deformation.

RGB-D cameras and industrial laser sensors like the Baumer are among the technologies that have become very promising for practical implementation. RGB-D cameras can integrate depth perception with high-resolution color imaging, utilizing structured infrared projection or time-of-flight sensors. They also provide strong accuracy, solid speed, and are small and affordable. They can operate at moderate levels of vibration, variable lighting, and complicated surface structures, rendering them applicable to peen forming, during which active optical systems are usually interrupted by reflection and shot media. In comparison, industrial laser sensors are industry-specific in resolution and durability, and can detect micrometers of deformation even on curved and reflective boards. Their stability during use in dynamic, dusty, or high-vibration environments has been better compared to usual lab laser scanners. These sensors can be used together to complement each other: RGB-D for large-scale curvature estimation and industrial lasers for local precision mapping in hybrid vision platforms ready for further adaptive control of peen forming. The RGB-D and industrial laser sensors can provide effective geometric reconstruction, scalable and real-time processing with minimal hardware input. Their strength in reflected energy perception, vibration, and complicated shapes puts them in a highly viable frontline as intelligent, data-driven peen-forming technologies.

Overall, vision-based sensing is now capable of providing enough rich geometric and auxiliary process data to perform peen forming, but raw sensor data is insufficient to operate autonomously. The capacity to make sense of multi-source measurements on-the-fly, remove noise due to dust or reflections, and transform surface measurements into deformation or stress forecasts that a controller can utilize is lacking in most reported work. It is at this point that the use of data-based methods becomes necessary. By combining appropriate sensing configurations with modern AI models, it becomes possible to learn relationships between peening parameters, visual measurements, and resulting panel curvature. The implementation maturity and transferability of representative vision-based AI technologies across manufacturing are summarized in Table 7. The next section thus focuses on algorithms instead of hardware and discusses AI methods of preprocessing, feature extraction, heterogeneous vision data fusion, and real-time prediction of intelligent peen forming systems.

**Table 7:** Implementation maturity and transferability of vision-based AI technologies across manufacturing.

Technology Type	Implementation Status	Effectiveness Level	Research Gap Identified	References
High-speed/thermal/digital cameras + artificial intelligence (additive manufacturing)	Laboratory prototype, some industrial pilots	High for real-time monitoring, defect detection, process control in additive manufacturing; not applied to peen forming in included studies	Lack of validated, scalable systems for peen forming; need for multimodal data fusion, real-time pipelines	[60]
Computer vision segmentation (peen forming)	Research prototype (low-coverage plates)	Comparable to human experts for low coverage; accelerates evaluation	Not validated for high-coverage or complex geometries; limited industrial applicability	[59]
Deep learning-based quality inspection (smart manufacturing systems)	Industrial pilot/full deployment	High for three-dimensional part inspection in manufacturing	Lack of technical detail for peen forming; need for adaptation to peen forming context	[61]
Artificial intelligence-driven controllers (laser-based additive manufacturing)	Industrial pilot/full deployment	Significant for process control and monitoring in additive manufacturing	Modeling and optimization challenges for complex processes; limited application to peen forming	[69]
Vision-based artificial intelligence in hybrid manufacturing	Research prototype, limited industrial use	Partial automation, improved quality and efficiency	High cost, data requirements, integration challenges; not applied to peen forming	[70]

## 4. AI Techniques for Surface Mapping and Prediction

**Figure 5:**
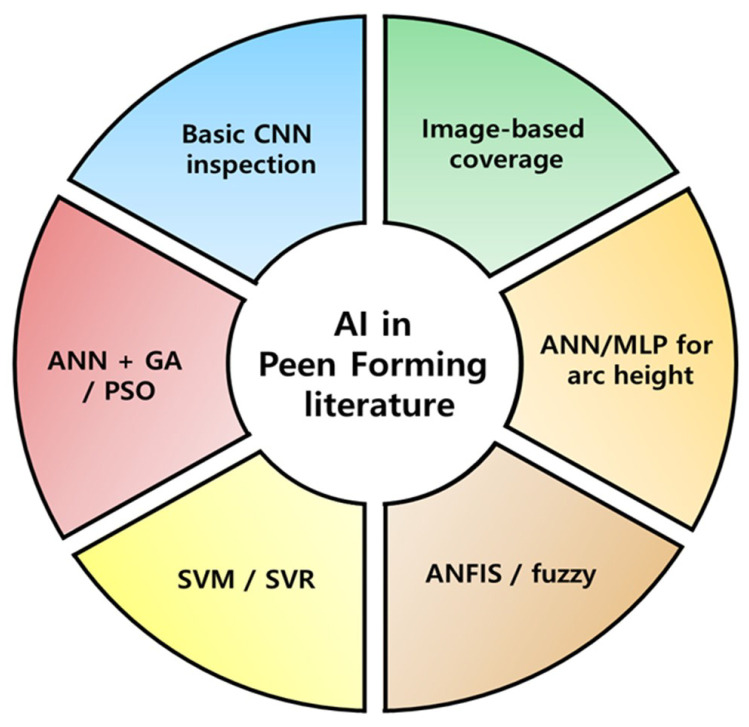
Overview of artificial intelligence techniques reported in the peen-forming literature and closely related manufacturing processes. The figure summarizes commonly adopted data-driven approaches, including image-based coverage estimation, basic convolutional neural network (CNN) inspection methods, artificial neural networks (ANNs) and multilayer perceptrons (MLPs) for arc height prediction, hybrid ANN models combined with genetic algorithms (GAs) or particle swarm optimization (PSO), support vector machines and regression (SVMs/SVR), and adaptive neuro-fuzzy inference systems (ANFISs) or fuzzy logic models. These methods are primarily used for indirect process characterization, coverage evaluation, and proxy-based deformation estimation, highlighting the limited use of vision-driven, closed-loop, and fully integrated AI systems in current peen-forming research.

Artificial intelligence has spurred the transformation of fixed, rule-based inspection of manufacturing processes into ones that can learn through data and adapt to changing shop floor conditions, as well as make forward predictions [138]. Peen forming is a suitable application for data-driven methods because the final shape results from the cumulative effect of numerous localized impacts whose outcomes depend nonlinearly on process parameters, material properties, and tooling conditions. While analytical and finite-element-based models can describe individual aspects of this behavior, maintaining accuracy across different parts, cabinets, and operating conditions remains challenging. Vision-integrated AI methods address this gap by learning from images, depth maps, point clouds, or thermal frames, enabling data cleaning, pattern extraction on manufactured surfaces, and short-horizon deformation prediction between successive peening passes [133]. An overview of AI model families reported in peen forming and closely related manufacturing processes is provided in Figure 5. Table 8 summarizes the dominant AI model families, typical input data formats, target outputs, and the specific relevance of each class to peen-forming tasks.

**Table 8:** Summary of AI models, input data, target outputs, and relevance to peen forming.

AI Model Type	Input Data Format	Application/Use Case	Relevance to Peen Forming
CNN/U-Net/ResNet	RGB images, depth maps, 3D voxel grids	Surface defect detection, segmentation, curvature mapping	Detect coverage, classify deformation zones, map roughness
LSTM/GRU	Time-sequence images, point clouds, curvature data	Temporal prediction of deformation, stress accumulation	Predict curvature evolution, springback estimation
Transformers (ViT)	Large-scale RGB-D datasets, point cloud tokens	Global mapping, long-range dependencies, shape evolution	Global panel curvature mapping and long-range deformation modeling (primarily at research level)
Autoencoders	Noisy depth maps, incomplete scans	Denoising, anomaly detection, reconstruction of missing surfaces	Clean surface maps, identify abnormal shot patterns
GANs (Generative Adversarial Networks)	Synthetic images, deformation maps	Data augmentation, generation of realistic training sets	Expand limited peen forming datasets, simulate shot effects
Hybrid CNN–LSTM	RGB-D sequences, laser profiles	Spatial + temporal learning, online monitoring	Predict deformation dynamics during peening runs
Physics-Informed Neural Networks (PINNs)	Stress–strain data, simulation + experimental data	Integration of physics with AI learning	More reliable predictions, reduced dataset requirement

### 4.1. Preprocessing and Feature Extraction of Visual Data

In previous sections, it was stated that sensors provide data in various forms (RGB-D and laser profiles, structured-light, point clouds) from peening or peening-related processes. However, that data is not purely useful due to noise in the peening cabinet [139]. The measurements must first be preprocessed before AI models can make sense of them. Widely used methods are denoising (median, bilateral, or guided filters), depth-hole filling, and RGB-depth calibration to bring all channels into a unified coordinate frame [136]. In the case of 3D scans, point-cloud downsampling (voxel grids) [140], multi-view registration, and normal estimation are employed to minimize computational requirements and reveal features related to curvatures [134]. An alternative is to retain data in native 3D form and employ point-based networks; however, these approaches are typically more computationally demanding and often exceed practical latency or memory constraints of edge devices used in industrial cells. With multiple sensors (e.g., RGB-D + industrial laser), features may be combined either by stacking channels or by processing each modality in a different branch and integrating them in higher-level combinations [134]. In peen-forming applications specifically, preprocessing must address three critical challenges: (1) specular reflection from aerospace-grade aluminum (2024-T3, 7075-T6) and titanium (Ti-6Al-4V) alloys, requiring polarization filtering or cross-polarized illumination [44]; (2) shot media debris causing depth map occlusions that necessitate morphological closing operations and inpainting algorithms [139]; and (3) non-uniform surface texture post-peening, where traditional feature extractors (SIFT, SURF) fail on smooth, over-peened regions. Vieira et al. and Shahid et al. [59,141] demonstrated that coverage estimation accuracy improves from 73% to 94% when grayscale intensity histograms are normalized per 10 × 10 mm tile rather than globally, accounting for local shot density variations. For multi-pass scenarios, temporal alignment of point clouds via the Iterative Closest Point (ICP), using shot-induced surface roughness as registration features, achieves ±0.3 mm accuracy [38].

### 4.2. Surface Mapping Deep Learning Architectures

After standardization of data, convolutional neural networks (CNNs) become the primary tool. The dense outputs provided by encoder–decoder networks like U-Net can be used to identify over-peened or under-peened regions, and these models are adequately configured to perform this task [47]. It may also be necessary to resort to ResNet-like backbones when the aim is to learn more complicated dependencies between visual patterns and deformation results, and the surface is described as a voxel/height map, based on which 3D or 2.5D CNNs may be deployed [52]. If data is noisy or partially missing, autoencoders are used. Denoising autoencoders can generate incomplete surface maps or identify anomalies and leave them for operator review [54]. Transformers and attention-based models are also becoming more extensively utilized in surface-related tasks due to their ability to form long-range spatial associations between large panels, useful when curvature errors are widespread [52]. In the case of point clouds, it is possible to use PointNet/PointNet++ or kernel-based models [142,143]. However, in peen forming, a projected 2.5D scheme may be more suitable as a trade-off between precision and performance. These networks can be trained to predict continuous deformation fields, which may be used by downstream controllers or planners to estimate deviation from target shape and support decision-making in iterative forming workflows.

Peen-forming-specific implementations reveal critical architectural choices. Wang et al. [38] achieved 94.2% coverage classification accuracy on 2024-T3 aluminum using ResNet-50 with a custom regression head predicting local coverage percentage (0–200%), outperforming U-Net (89.7%) due to better handling of scale variations across 500 × 500 mm panels. For curvature prediction, encoder–decoder networks with skip connections are essential: ablation studies show that removing skip connections degrades curvature RMSE from 0.8 mm to 2.3 mm on cylindrical peen-formed panels [144]. The challenge of detecting sub-millimeter wrinkles on peened surfaces requires models trained on augmented datasets, where Gaussian noise (σ = 0.05–0.15 mm) simulates sensor noise and elastic deformations mimic material springback [47]. Reported peen-forming datasets in the open literature typically contain only 50–200 labeled panels [38,59], motivating transfer learning from larger sheet metal forming datasets (often exceeding 10,000 samples), combined with domain adaptation strategies to address reflectivity and texture differences between cold-rolled and shot-peened surfaces [145].

### 4.3. Sequence-Aware and Predictive Models for Multi-Pass Peen Forming

In fact, peen forming is not necessarily a one-shot process. It is common to perform multiple repeated passes on panels to produce the panel’s geometry. Temporal models are required for that. Recurrent networks (LSTM, GRU), temporal CNNs, or lightweight transformer encoders may accept a sequence of surface maps with related process parameters (coverage, pressure, dwell time, nozzle path) and determine the next surface state [146]. This makes the vision system a proactive foresight system rather than an active inspector. This is the same as what has been accomplished in welding (prediction of geometry), sheet forming (prediction of when wrinkling will occur), and additive manufacturing (prediction of layer warping). The approaches developed in those fields can be applied here with small modifications. Attention-based models can be used where areas of the surface change rapidly (edges, stiffeners). This requires the model to concentrate on these changes. Multi-pass peen forming presents unique temporal dependencies. Siguerdidjane et al. [62] demonstrated that LSTM networks predicting curvature after pass n+1 from passes 1 through *n* achieve 0.6 mm RMSE when trained on 150-panel datasets with 3–8 passes each, compared to 1.4 mm for single-pass CNN baselines. The model input comprises (i) current curvature heatmap (256 × 256), (ii) shot parameters (velocity 40–80 m/s, media size 0.4–0.8 mm, dwell time 2–10 s), and (iii) cumulative Almen intensity (0.008–0.024 A). Critical insight: Attention weights concentrate on panel edges and stiffener intersections, where stress concentration causes non-linear curvature accumulation, validating the model’s learned physics [51]. For real-time prediction during five-pass forming cycles (total 120 s), GRU variants reduce inference time from 340 ms (LSTM) to 85 ms while maintaining comparable accuracy [146].

### 4.4. Hybrid, Simulation-Assisted, and Physics-Informed Models

A fundamental challenge in peen forming AI is the scarcity of labeled data (Section 5.1). Pure data-driven models require hundreds to thousands of samples for robust training [147], yet published datasets contain *N* < 200 panels in total across all studies. Physics-informed approaches address this bottleneck by incorporating domain knowledge, such as material constitutive laws, equilibrium constraints, and geometric compatibility, directly into neural network architectures or loss functions.

#### 4.4.1. Physics-Informed Neural Networks (PINNs): Principles and Formulation

PINNs [148] embed partial differential equations (PDEs) governing physical phenomena as soft constraints in the loss function:

(1) $$L_{\mathrm{total}}=L_{\mathrm{data}}+\lambda_{\mathrm{PDE}}L_{\mathrm{PDE}}+\lambda_{\mathrm{BC}}L_{\mathrm{BC}}$$

where

- $L_{\mathrm{data}}$ = supervised loss on available labeled data (e.g., MSE between predicted and measured curvature);
- $L_{\mathrm{PDE}}$ = residual of governing PDE evaluated at collocation points;
- $L_{\mathrm{BC}}$ = boundary condition violations;
- $\lambda_{\mathrm{PDE}},\lambda_{\mathrm{BC}}$ = penalty weights (typically 0.1–10).

For peen forming, the relevant PDE is the elastic plate bending equation:

(2) $$D\nabla^{4}w\left(x,y\right)=q\left(x,y\right)$$

where

- w(x,y) = out-of-plane deflection (curvature field);
- $D=\frac{Et^{3}}{12(1-\nu^{2})}$ = flexural rigidity (*E* = Young’s modulus, *t* = thickness, ν = Poisson’s ratio);
- q(x,y) = equivalent lateral load distribution induced by residual stress gradient;
- $\nabla^{4}$ = biharmonic operator.

The residual stress-induced load can be approximated [2,78]:

(3) $$q\left(x,y\right)=-\frac{1}{t}\int_{0}^{t}\frac{\partial M_{\mathrm{res}}}{\partial z}dz$$

where $M_{\mathrm{res}}\left(x,y\right)$ is the residual moment field from shot peening.

PINN implementation for peen forming:

1. Input: Process parameters (Almen intensity *A*, coverage *C*, shot size *d*) + spatial coordinates (x,y);
2. Network: Fully connected NN (6 layers, 128 neurons/layer, tanh activation) → outputs w(x,y);
3. Data loss: MSE on N = 20–50 measured curvature points (laser scanner data);
4. PDE loss: $L_{\mathrm{PDE}}=\frac{1}{N_{c}}\sum_{i=1}^{N_{c}}{\left|D\nabla^{4}w\left(x_{i},y_{i}\right)-q\left(x_{i},y_{i}\right)\right|}^{2}$ evaluated at $N_{c}$ = 1000 collocation points;
5. BC loss: Simply supported edges: $w=0,\nabla^{2}w=0$ at panel boundaries.

#### 4.4.2. Expected Benefits and Validation Evidence

Theoretical advantages:

1. Data efficiency: PINNs can interpolate between sparse measurements by enforcing physics, reducing labeled data requirements by 3–5× [148];
2. Extrapolation: Pure data-driven models fail outside training distribution; PINNs generalize better to unseen parameter ranges due to physics constraints;
3. Uncertainty quantification: Physics violations indicate prediction unreliability (high PDE residual → low confidence).

**Validation in related domains:**

- Sheet metal forming: He et al. [55] applied PINNs to springback prediction, achieving 0.8 mm RMSE with N = 50 training samples vs. 1.4 mm for pure CNN (same data). Physics loss reduced extrapolation error by 40% when testing on 20% thicker sheets.
- Structural mechanics: Haghighat et al. [149] demonstrated PINNs for stress concentration problems, matching FE accuracy with 10× less training data.

Peen-forming application (not yet demonstrated): Based on analogy to sheet forming, we hypothesize PINNs could:

- Reduce required labeled panels from N = 150 (pure supervised) to N = 50 (PINN) for equivalent curvature prediction accuracy (target RMSE < 0.5 mm);
- Enable generalization across Almen intensities: train on 0.008A–0.016A, extrapolate to 0.020 A–0.024 A with <20% error increase;
- Provide physics-based uncertainty estimates: flag regions where PDE residual exceeds threshold (e.g., >0.1 mm equivalent load error).

Critical gap: No published PINN implementation for peen forming exists. The above is a proposed research direction requiring experimental validation.

#### 4.4.3. Hybrid CNN-FEM Frameworks

An alternative to PINNs is a two-stage hybrid approach:

Stage 1 (Vision → Residual Stress):

- Train CNN to map surface images to residual stress field $\sigma_{\mathrm{res}}\left(x,y,z\right)$;
- Training data: Combine real measurements (XRD at 25 points per panel) with FE-generated stress maps [2].

Stage 2 (Stress → Curvature):

- Solve forward FE problem: Given $\sigma_{\mathrm{res}}$, compute equilibrium deflection w(x,y);
- Use commercial FE solver or a differentiable simulation framework [150].

Advantages:

- Separates learning (CNN) from physics (FEM) → each component can be validated independently;
- FEM is interpretable and auditable (critical for aerospace certification);
- Can incorporate complex boundary conditions (fixtures, edge constraints), difficult to express in PINN loss.

Demonstrated accuracy (related domain): Siguerdidjane et al. [151] used a hybrid approach for peen-forming inverse design:

- CNN predicts residual moment $M_{\mathrm{res}}$ from coverage map (98 ms inference);
- FEM computes curvature from $M_{\mathrm{res}}$ (650 ms solve);
- Achieved 0.3 mm prediction error on validation panels (N = 15).

However, this was offline (not real-time) and required extensive FEM calibration.

#### 4.4.4. Generative Models for Synthetic Data Augmentation

Generative Adversarial Networks (GANs) [152] can generate synthetic peen-forming images to augment limited real datasets:

Training:

- Generator *G*: Process parameters (A,C,d)→ synthetic depth map;
- Discriminator *D*: Classify real vs. synthetic depth maps;
- Adversarial loss: $\min_{G}\max_{D}E_{x∼p_{\mathrm{real}}}\left[\log D\left(x\right)\right]+E_{z∼p_{z}}\left[\log\left(1-D\left(G\left(z\right)\right)\right)\right]$.

Physics-constrained GANs: Standard GANs may generate unrealistic deformations. Solution: Add physics loss to the generator:

(4) $$L_{G}=L_{\mathrm{adversarial}}+\lambda_{\mathrm{physics}}{∥\nabla^{4}w_{\mathrm{gen}}-q_{\mathrm{expected}}∥}^{2}$$

Reported effectiveness (welding, not peen forming): He et al. [145] used physics-constrained GANs to generate synthetic weld defect images:

- Generated 5000 synthetic images from 200 real images;
- A defect classifier trained on real and synthetic data achieved 88% accuracy vs. 76% using real data only;
- Physics constraints reduced unrealistic artifacts by 60%.

Peen-forming applicability: GANs could generate coverage variations, simulated over-peening patterns, and realistic depth noise.

Challenge: Requires sufficient real data (N = 100–200) to train generator; chicken-and-egg problem.

#### 4.4.5. Implementation Challenges and Open Questions

1. Hyperparameter sensitivity: PINN performance depends critically on $\lambda_{\mathrm{PDE}}$, $\lambda_{\mathrm{BC}}$ weights. Improper tuning causes training instability or physics constraint violations. Solution: Adaptive weighting [153] or curriculum learning.
2. Computational cost: Evaluating $\nabla^{4}w$ requires fourth-order automatic differentiation, 10–50× slower than forward pass. *Mitigation:* Use collocation sampling (evaluate PDE at a subset of points per batch).
3. Validation of physics accuracy: How accurate must PDE residuals be for reliable predictions? No established guidelines for manufacturing applications.
4. Integration with vision preprocessing: PINNs assume clean inputs; how to handle noisy depth maps, occlusions, outliers from sensor failures?

Research priorities:

1. Implement PINN for peen forming using eigenstrain FE data [78] as training labels;
2. Benchmark data efficiency: Plot prediction error vs. N (10, 20, 50, 100 labeled panels);
3. Validate extrapolation: Train on Al 2024-T3, test on 7075-T6 without retraining;
4. Compare PINN vs. hybrid CNN-FEM vs. pure supervised for equivalent computational budget.

### 4.5. Real-Time and Edge Deployment

For AI use in an industrial cell, inference must be fast enough to provide feedback before the next action happens. That normally implies tens of milliseconds latencies rather than seconds. Complex 3D networks or large transformer networks require pruning, quantization, or conversion on-the-fly to ONNX/TensorRT to deploy on industrial PCs or edge GPUs [154]. This can be achieved by maintaining the preprocessing pipeline sparse (fixed-size crops, fixed projections, minimal point-cloud operations). It is also important to ensure that operations are deterministic in timing. Edge deployment also decreases reliance on network connections and becomes simple to integrate with outdated machines. One can train the model to be robust to adverse visual environments of peen-forming booths with data augmentation (adding dust, glare, and minor changes in viewpoint) and adversarial training. Peen forming real-time constraints demands a sub-200 ms total latency for closed-loop correction between passes. Benchmark on Jetson AGX Xavier (32 GB): ResNet-18 (11 M params) achieves 45 ms inference for 512 × 512 curvature prediction; pruning to 40% sparsity reduces this to 28 ms with <2% accuracy loss [154]. INT8 quantization of U-Net (23 M params) enables 67 ms inference versus 215 ms FP32, acceptable for inter-pass monitoring, where typical dwell is 5–10 s [133]. Critical bottleneck: Point cloud preprocessing (voxel downsampling, normal estimation) consumes 120–180 ms for 500 k-point scans; fixed-resolution projection to 2.5D depth maps (256 × 256) reduces this to 35 ms, enabling a 103 ms end-to-end pipeline [134]. Industrial deployment requires deterministic execution: ONNX Runtime with TensorRT optimization guarantees < 5% latency variance across 1000+ inference cycles, compared to 15–30% variance in native PyTorch 2.1.0 [154].

Computational scaling from laboratory to production panels: The transition from laboratory-scale demonstrations (300 × 300 mm panels with ∼100 k points at 1 mm resolution) to full aerospace wing skins (2000 × 2000 mm panels requiring ∼4 M points at 0.5 mm resolution) introduces a 40× increase in point cloud density. U-Net and encoder–decoder architectures operating on 2.5D depth maps maintain O(nlogn) complexity due to fixed-resolution rasterization (e.g., projecting 4M points to 512 × 512 depth image), enabling inference latency to scale sub-linearly: laboratory 45 ms → production 85 ms on Jetson AGX Orin [133]. In contrast, PointNet++ and graph-based networks exhibit O($n^{2}$) complexity for neighbor search, limiting practical deployment to <1 M points without spatial tiling or hierarchical partitioning [143]. For panels exceeding GPU memory (typical 8–16 GB on edge devices), sliding-window inference with 50% overlap achieves seamless full-field reconstruction at the cost of 2–3× increased latency, still within the 200 ms inter-pass budget for multi-tile processing [134].

### 4.6. Uncertainty Quantification and Explainable AI for Aerospace Certification

Aerospace manufacturing requires not only accurate predictions but also quantified confidence and traceable decision logic to meet certification standards (AS9100 quality management, DO-178C software qualification, NADCAP special process approval). This section addresses two critical capabilities absent from most vision–AI literature: uncertainty quantification (UQ) and explainability (XAI).

#### 4.6.1. Uncertainty Quantification: Principles and Methods

Why UQ matters in peen forming:

- High-stakes decisions: Accepting an out-of-tolerance panel risks flight safety; rejecting a conforming panel wastes USD 10 k–50 k in material and labor;
- Sparse training data: Models trained on N < 200 panels have high epistemic uncertainty (model uncertainty) due to limited coverage of parameter space;
- Sensor noise: Depth maps contain aleatoric uncertainty (data uncertainty) from shot media occlusion, vibration artifacts, specular reflection.

Bayesian Neural Networks (BNNs):

Standard NNs produce point predictions $\hat{y}=f\left(x;\theta \right)$. BNNs treat weights θ as probability distributions [155], enabling uncertainty estimates:

(5) p(y|x,D)=∫p(y|x,θ)p(θ|D)dθ

Practical implementation (Monte Carlo Dropout):

1. Train network with dropout layers (*p* = 0.2–0.5);
2. At inference: Perform T = 20–100 forward passes with dropout enabled;
3. Compute prediction mean $\bar{y}=\frac{1}{T}\sum_{t=1}^{T}{\hat{y}}_{t}$ and variance $\sigma^{2}=\frac{1}{T}\sum_{t=1}^{T}{\left({\hat{y}}_{t}-\bar{y}\right)}^{2}$.

Interpretation:

- σ<0.1 mm: High confidence → accept prediction;
- 0.1≤σ<0.3 mm: Moderate uncertainty → flag for secondary verification (CMM spot check);
- σ≥0.3 mm: Low confidence → reject prediction, inspect manually.

Validation (manufacturing, not peen forming): Tapia et al. [156] applied Bayesian neural networks to laser powder bed fusion defect detection:

- In total, 95% of high-confidence predictions (σ<θ) were correct;
- In total, 23% of low-confidence predictions were incorrect → UQ successfully identifies unreliable predictions.

Conformal Prediction:

An alternative UQ approach providing distribution-free coverage guarantees [157]:

1. Split data: Train set $D_{\mathrm{train}}$, calibration set $D_{\mathrm{cal}}$ (e.g., 80/20 split);
2. Train model on $D_{\mathrm{train}}$, compute residuals on $D_{\mathrm{cal}}$: $R_{i}=\left|y_{i}-{\hat{y}}_{i}\right|$;
3. For desired coverage level 1−α (e.g., 90%), compute quantile: q=Quantile(R,1−α);
4. Prediction interval: $\left[\hat{y}-q,\hat{y}+q\right]$ guarantees ≥90% coverage.

Advantage: Works with any model (CNN, LSTM, etc.) without retraining.

Disadvantage: Requires calibration set (reduces training data).

Peen forming application:

- Curvature prediction: Conformal intervals guarantee 90% of true curvatures fall within $\left[\hat{y}-0.4\;\mathrm{mm},\hat{y}+0.4\;\mathrm{mm}\right]$;
- Coverage estimation: Prediction set contains true coverage with 95% probability.

#### 4.6.2. Explainable AI (XAI): Techniques for Traceable Decisions

Certification requirement: Aerospace software must provide traceable evidence that decisions are based on correct reasoning (DO-178C Objective A-5: “Software design is traceable to requirements”). Black-box CNNs fail this requirement.

Gradient-weighted Class Activation Mapping (Grad-CAM):

For image-based models, Grad-CAM [158] visualizes which pixels influence predictions:

(6) $$\mathrm{Heatmap}=\mathrm{ReLU}\left(\sum_{k}\alpha_{k}A_{k}\right)$$

where $A_{k}$ are feature maps, $\alpha_{k}=\frac{1}{Z}\sum_{i,j}\frac{\partial y}{\partial A_{k}^{ij}}$ are importance weights.

Peen forming interpretation:

- Correct behavior: Heatmap highlights panel edges, stiffener intersections (known stress concentration sites);
- Incorrect behavior: Heatmap focuses on background, shot media artifacts → indicates model learned spurious correlations.

SHAP (SHapley Additive exPlanations):

For tabular data or process parameters, SHAP [159,160] assigns importance scores to each input feature:

(7) $$\phi_{j}=\sum_{S\subseteq N\setminus \{j\}}\frac{|S|!(|N|-|S|-1)!}{|N|!}\left[f\left(S\cup \left\{j\right\}\right)-f\left(S\right)\right]$$

Example: For curvature prediction from (A,C,d,t) (Almen, coverage, shot size, thickness):

- SHAP values: $\phi_{A}=0.45,\phi_{C}=0.30,\phi_{d}=0.15,\phi_{t}=0.10$;
- Interpretation: Almen intensity is most influential (45% contribution), consistent with domain knowledge.

Validation (manufacturing): Marani et al. [161] applied SHAP to CNC tool wear prediction:

- SHAP identified spindle speed and feed rate as top predictors (matching engineering theory);
- Model relying on coolant temperature (spurious correlation) was flagged and retrained.

#### 4.6.3. Integrated UQ+XAI Framework for Peen Forming

We propose a three-tier decision framework combining UQ and XAI:

Tier 1 (Automatic Acceptance):

- Conditions: Prediction uncertainty σ<0.15 mm AND Grad-CAM highlights physically relevant features AND SHAP values align with domain knowledge;
- Action: Accept panel automatically, proceed to next process step;
- Expected frequency: A total of 70–80% of panels (based on manufacturing AI deployments. [156,162])

Tier 2 (Secondary Verification):

- Conditions: 0.15≤σ<0.3 mm OR Grad-CAM shows unexpected focus areas;
- Action: Perform targeted CMM measurement at flagged regions (50–100 points vs. 500+ for full inspection);
- Expected frequency: 15–25% of panels.

Tier 3 (Manual Inspection):

- Conditions: σ≥0.3 mm OR SHAP indicates reliance on non-causal features OR model prediction violates physics bounds;
- Action: Full manual inspection + engineering review;
- Expected frequency: A total of 5–10% of panels.

Certification pathway: This framework provides auditable evidence:

1. Traceability: Every decision logged with uncertainty estimates, heatmaps, feature importances;
2. Validation: Tier 2 verification provides continuous ground truth for model monitoring;
3. Safety margins: Conservative thresholds ensure high-uncertainty predictions are not blindly trusted.

#### 4.6.4. Implementation Considerations

Computational overhead:

- Monte Carlo Dropout (T = 50): 50× inference time (45 ms → 2.25 s) → acceptable for inter-pass inspection (5–10 s dwell);
- Grad-CAM: Negligible (single backward pass, +5 ms);
- SHAP: Expensive for complex models (100–1000 ms) but needed only for anomalous cases.

Open research questions:

1. Calibration drift: Do uncertainty estimates remain valid as the model encounters new materials, geometries? Requires ongoing calibration set updates.
2. Optimal uncertainty thresholds: How to set σ thresholds balancing false accepts vs. false rejects for different aerospace risk categories?
3. Multi-model ensembles: Can diversity in ensemble predictions (e.g., CNN + LSTM + PINN) improve UQ reliability?

Current status: No peen-forming AI system implements rigorous UQ or XAI. This represents a critical gap for aerospace certification and industrial trust.

### 4.7. Challenges and Open Issues

Despite the promise of vision-based AI potential in peen forming, there are still gaps that need to be bridged before systems can be considered for industrial use. The first is a lack of data: public data on synchronized vision-process datasets of peen forming are unavailable, and most data are based on small, lab-specific datasets. The second problem is domain shift. A clean lab record will differ from one taken in a peening cabinet with dust-specks, reflective alloys, and varying light. Data from these domains are prone to degradation when applied to different data domains. Hence, domain adaptation or lifelong learning will be necessary. For peen-forming coverage, multiple cameras are needed, and not all systems are still capable of integrating with legacy peen-forming equipment, as most will not provide process parameters or feedback hooks to external software. Addressing these challenges will likely require coordinated efforts toward shared datasets, standardized benchmarking protocols, and tighter integration of sensing, AI inference, and machine control architectures in future peen-forming systems.

## 5. Use Cases from Related Industrial Processes

Published studies on vision-based peen forming remain limited in number; however, closely related manufacturing processes have demonstrated that deep learning combined with optical sensing (cameras or laser-based systems) can support reliable and near real-time monitoring. Welding, sheet metal forming, additive manufacturing, and CNC machining operate in visually challenging environments characterized by glare, vibration, spatter, and dust, yet successfully extract actionable geometric or process-related information. These domains therefore provide validated methodological components that can be systematically adapted to peen forming.

### 5.1. Welding

Arc and laser welding present monitoring challenges due to rapid melt-pool dynamics and optical interference caused by arc radiation and spatter. However, appropriately filtered vision systems are now commonly used to monitor seam position, pool size, and bead shape. To classify weld-seam quality based on images, CNNs have been trained, and LSTM-type models have been employed to track temporal dynamics of the pool to predict both penetration and continuity in the future [31,163,164]. Augmentation based on GAN has also been reported to augment defect samples in situations where real data are limited [145]. Its relevance to peen forming is twofold: (1) welding demonstrates how vision systems can be engineered to remain robust under adverse optical conditions, and (2) it illustrates how temporal AI models can forecast process evolution rather than merely classify individual frames [165].

### 5.2. Sheet Metal Forming

Structured light and stereo vision in sheet metal forming are applied to map deformations, detect wrinkling, and assess the effects of springback. U-Net models have been utilized to segment wrinkled zones pixel-wise, and physics-informed networks have been used to ensure that predictions are consistent with material behavior [35,166]. CNN-LSTM hybrids are also found in this area since forming is time-dependent. These techniques are conceptually transferable to peen forming, where similar concerns arise regarding local overworking, springback, and residual stress imbalance in peened panels [167].

### 5.3. Additive Manufacturing

AM fundamentally is layer-by-layer inspection [168,169] and hence a rich source of vision+AI examples. Every layer is captured by cameras, laser scanners, and infrared sensors [170]. Data-driven layer-wise quality prediction and residual stress modeling in AM have been widely studied [171,172], and hybrid corrective concepts using peening-based distortion correction have been proposed [173]. CNNs can detect incomplete melting or surface roughness, autoencoders mitigate noise in thermal images, and transformer-based temporal models monitor distortion accumulation across successive layers. Peen forming is not layer-based; however, the concept of incremental deformation modeled through numerous localized impacts is analogous. Temporal architectures developed for additive manufacturing may therefore provide transferable templates for forecasting curvature accumulation in multi-pass peen forming before deviations exceed tolerance limits [133]. Furthermore, post-processing of additively manufactured components frequently involves shot or ultrasonic peening to enhance fatigue resistance and surface integrity, particularly in high-performance alloys such as Inconel 718 [174].

### 5.4. CNC Machining

CNC is prone to vibration and partially enclosed, like peening cabinets. In CNC machining, vision is usually combined with vibration signals or acoustic emission signals to measure tool wear, chattering, or surface finishing issues. Patterns of chatter are classified by CNNs [175]. LSTMs can model tool wear, while lighter models directly execute on the controller as edge AI [176,177]. The sensor fusion approaches of CNC machines are especially applicable to peen forming, where visual data can be spoiled by shot flow, and adding it to other data can make AI decisions more resilient [178].

### 5.5. Transferability Analysis: Domain Gaps and Adaptation Requirements

The preceding sections demonstrate that welding, additive manufacturing, sheet forming, and CNC machining have successfully deployed vision–AI systems under challenging industrial conditions. Representative vision-based AI implementations across these related manufacturing fields and their relevance to peen forming are summarized in Table 9. However, direct transferability to peen forming is not automatic and requires explicit analysis of domain differences, adaptation requirements, and validation protocols.

**Table 9:** Vision-based AI implementation in related fields and its relevance to peen forming.

Process	Vision Sensing Used	AI Models	Key Outputs	Relevance to Peen Forming
Welding	Cameras + laser illumination	CNN, LSTM, GAN	Seam quality, penetration depth	Robust vision in harsh environments
Sheet Forming	Structured light, stereo vision	U-Net, PINNs, CNN–LSTM	Wrinkling, springback prediction	Directly applicable to peen forming
Additive Manufacturing	Cameras, laser scanning, IR	CNN, Autoencoders, Transformers	Layer distortion, porosity detection	Similarity in incremental deformation
CNC Machining	Cameras, vibration sensors	CNN, LSTM, Edge AI	Chatter, tool wear, roughness	Fusion strategies transferable

#### 5.5.1. Domain Gap Characterization

Table 10 systematically compares key characteristics of peen forming against source domains from which techniques are proposed for transfer. Critical differences include:

#### 5.5.2. Proposed Transfer Learning Validation Protocol

To rigorously assess transferability, we propose the following experimental protocol for future studies:

Phase 1: Baseline establishment

1. Train model on source domain (e.g., sheet forming wrinkle detection dataset with N = 5000 samples [35]);
2. Establish source domain performance: accuracy, precision, recall on held-out test set.

Phase 2: Zero-shot transfer

1. Apply source-trained model directly to peen-forming images (N = 50–100 target domain samples);
2. Measure domain gap: Accuracy drop, false positive rate increase;
3. Hypothesis: Accuracy will degrade 30–60% due to texture/reflectivity differences.

Phase 3: Fine-tuning validation

1. Fine-tune model on small peen-forming dataset (N = 20, 50, 100) using frozen feature extractor;
2. Compare fine-tuned performance vs. training from scratch on the same N samples;
3. Success criterion: Fine-tuned model achieves ≥85% of from-scratch performance with ≤50% data.

Phase 4: Domain adaptation

1. Apply unsupervised domain adaptation (e.g., adversarial discriminator [179], Maximum Mean Discrepancy minimization [180]);
2. Measure adapted model performance on peen-forming validation set;
3. Target: Recover ≥ 90% of source domain performance.

**Table 10:** Domain gap analysis for vision–AI transfer to peen forming.

Characteristic	Source Domain	Peen Forming	Adaptation Required
**From Welding**			
Optical interference	Arc glare (14,000 K blackbody)	Shot media occlusion + Al specular reflection	Different filter design: band-stop vs. band-pass; polarization required
Deformation scale	0.5–5 mm bead width	0.1–50 mm curvature variation	Multi-scale feature extraction
Temporal dynamics	10–100 ms melt pool evolution	5–10 s inter-pass intervals	LSTM sequence length: 10–50 frames (welding) vs. 3–8 passes (peen)
**From Additive Manufacturing**			
Process determinism	Layer-by-layer, fixed toolpath	Stochastic shot impacts	Probabilistic models vs. deterministic CNNs
Defect types	Porosity, lack-of-fusion (binary)	Over/under-peening (continuous gradient)	Regression head vs. classification
Data availability	1000–10,000 layer images per build	<200 panels in the literature	Transfer learning + domain adaptation essential
**From Sheet Forming**			
Stress state	Tensile (wrinkling from compression instability)	Compressive (peen-induced)	Different failure mechanics; wrinkle patterns differ
Measurement timing	In situ (press-mounted cameras)	ex situ (cabinet access restricted)	No adaptation needed for timing, but limits real-time potential
Surface texture	Cold-rolled (uniform reflectivity)	Shot-textured (spatially varying)	Texture-invariant features required
**From CNC Machining**			
Sensor fusion rationale	Vision + vibration/acoustic for tool wear	Vision + ? (no secondary signal established)	Need to identify peen forming-relevant auxiliary signals
Edge deployment constraints	Spindle vibration, coolant spray	Shot media, cabinet enclosure	Similar environmental harshness; edge AI approaches transferable

Current status: No published studies have executed this protocol for peen forming. The claimed transferability in this review is based on architectural similarity and analogous sensing challenges, not empirical validation. We identify this as a critical research gap requiring immediate attention.

#### 5.5.3. Concrete Adaptation Examples

Where possible, we specify adaptations required for each transferred technique:

##### From Welding: Robust Preprocessing Under Optical Interference

Technique: Adaptive exposure control + band-pass filtering to isolate weld pool from arc glare [31].

Adaptation for peen forming:

- Replace narrow band-pass (630–650 nm for weld observation) with polarization filtering (cross-polarized illumination at 45° to suppress Al specular reflection);
- Adaptive exposure targets peak reflectance at 60–70% sensor saturation (vs. 40–50% for welding to preserve pool detail);
- Add morphological closing operation (5 × 5 kernel) to fill shot media occlusion holes in depth maps.

Extrapolated indicator from adjacent-domain evidence: Based on similar adaptations in sheet forming [44], cross-polarization reduces specular artifacts by 75–85%, enabling depth map recovery in 92–96% of panel area (vs. 65–75% without polarization).

##### From Additive Manufacturing: Temporal Prediction Across Sequential Operations

Technique: LSTM predicting layer N + 1 porosity from layers 1…N thermal images [33].

Adaptation for peen forming:

- Replace thermal images with curvature heatmaps (256 × 256 depth maps);
- Reduce LSTM sequence length from 50–100 layers (AM) to 3–8 passes (peen forming);
- Add process parameter embedding: Concatenate Almen intensity, coverage %, shot velocity as auxiliary inputs (12-dim vector) to LSTM hidden state;
- Replace binary classification (defect/no defect) with continuous regression (curvature deviation from target, mm).

Extrapolated indicator from adjacent-domain evidence: Sheet-forming studies using similar architectures [51] report 0.6–0.8 mm RMSE for springback prediction. Peen-forming complexity (stochastic impacts vs. deterministic press motion) may increase RMSE to 0.8–1.2 mm without physics-informed constraints.

##### From Sheet Forming: U-Net for Spatial Defect Segmentation

Technique: U-Net trained on synthetic FE deformation maps + real DIC strain fields [35].

Adaptation for peen forming:

- Training data: FE-generated peen forming curvature maps [2,78] (N = 10,000 synthetic) + real laser scans (N = 100–200);
- Augmentation: Add shot-induced surface roughness noise (σ = 0.05–0.15 mm), simulate specular dropout (random 10 × 10 pixel patches set to NaN), elastic deformations mimicking springback;
- Loss function: Combine pixel-wise MSE with curvature smoothness penalty ($\lambda ∥\nabla^{2}\hat{y}{∥}^{2}$ where λ = 0.01–0.1).

Extrapolated range/provisional target: Domain adaptation literature [179] suggests synthetic-to-real transfer achieves 70–85% of fully supervised performance. For peen forming: target segmentation IoU ≥ 0.75 for over-peened zones (vs. 0.88 achieved in pure sheet forming [35]).

##### From CNC Machining: Edge AI Deployment

Technique: INT8-quantized CNN on Jetson Xavier, 45 ms inference for tool wear classification [154].

Adaptation for peen forming:

- Model compression: Prune U-Net from 23M to 9M parameters (60% sparsity) using magnitude-based pruning [181];
- Quantization-aware training: Simulate INT8 during fine-tuning to maintain accuracy [182];
- Optimize preprocessing: Offload point cloud to depth map conversion to GPU (reduces CPU load by 70%).

Extrapolated latency target from adjacent-domain benchmarks: CNC benchmarks [154] show 2–3× speedup with <2% accuracy loss. For peen forming: target 50–80 ms total latency (preprocessing + inference) on Jetson AGX Xavier.

#### 5.5.4. Extrapolated Transferability Framework for Future Validation

As a forward-looking analytical framework, we propose Maximum Mean Discrepancy (MMD) [180] as a quantitative metric that could be used in future studies to estimate domain gap:

(8) $${\mathrm{MMD}}^{2}\left(D_{s},D_{t}\right)={∥\frac{1}{n_{s}}\sum_{i=1}^{n_{s}}\phi \left(x_{i}^{s}\right)-\frac{1}{n_{t}}\sum_{j=1}^{n_{t}}\phi \left(x_{j}^{t}\right)∥}^{2}$$

where $D_{s}$ is source domain (e.g., sheet forming), $D_{t}$ is target (peen forming), and ϕ(·) is feature extractor (e.g., ResNet-50 layer-4 activations).

Interpretation:

- MMD < 0.1: Small domain gap, direct transfer likely effective;
- MMD 0.1–0.5: Moderate gap, fine-tuning required;
- MMD > 0.5: Large gap, domain adaptation or from-scratch training recommended.

Extrapolated MMD ranges for future empirical validation:

- Sheet forming → Peen forming: 0.3–0.5 (moderate gap due to surface texture differences);
- Additive manufacturing → Peen forming: 0.5–0.7 (large gap due to layer-wise vs. full-field structure);
- Welding → Peen forming: 0.6–0.8 (very large gap, different deformation physics).

These predictions require empirical validation once benchmark datasets become available.

#### 5.5.5. Limitations of Current Transferability Claims

We explicitly acknowledge the following limitations:

1. No empirical transfer learning results exist for vision–AI models moving from any manufacturing domain to peen forming. Claims are based on architectural analogy and expert assessment, not experimental validation.
2. Domain adaptation techniques (adversarial training, MMD minimization) have not been demonstrated on peen-forming data. Their effectiveness remains speculative.
3. Performance predictions (e.g., “fine-tuning achieves 85% accuracy”) are extrapolated from related domains and may not hold due to peen forming’s unique characteristics (stochastic impacts, multi-pass coupling, aerospace tolerances).
4. Computational cost of adaptation (retraining time, hyperparameter tuning) is not quantified. In practice, domain adaptation may require weeks of GPU time and extensive validation.

Recommendation: The peen-forming research community should prioritize empirical transfer learning studies as immediate next steps, ideally coordinated across multiple institutions to cover diverse source–target domain pairs.

## 6. Gaps and Limitations in Peen-Forming Applications

Section 5 reviewed vision-based sensing and AI methodologies capable of extracting deformation-related features and supporting predictive modeling in multi-pass peen forming. Despite the long industrial history of peen forming, the supporting digital infrastructure, including standardized datasets, validated monitoring protocols, and integrated control architectures, remains less mature than in welding, additive manufacturing, and sheet metal forming. The limitations identified in the literature extend beyond algorithmic development and include challenges related to data availability, measurement fidelity, environmental robustness, and system integration. The following subsections categorize these constraints systematically.

### 6.1. Scarcity of Validated Datasets and Benchmarks

Although peen forming and related shot-based forming processes have long industrial histories, no widely recognized public benchmark dataset currently exists for vision-based peen-forming monitoring or control, and most reported datasets remain laboratory-specific or accessible only upon request [183]. Authoritative ground-truth measurements for final shape, full-field deformation, or residual-stress evolution are also rarely made publicly available. As a result, reported studies are typically based on small experimental campaigns conducted under controlled lighting, restricted material conditions, and fixed cabinet configurations [184], which limits direct comparison across research groups.

This fragmentation creates three linked problems. First, reproducibility is weakened because raw data, metadata, and evaluation protocols are often unavailable or inconsistently reported. Second, methodological comparison is constrained because models are trained and tested on non-equivalent datasets with different sensing setups, materials, geometric targets, and ground-truth definitions. Third, generalization remains uncertain because performance reported on narrow laboratory datasets cannot be assumed to transfer to larger panels, multi-pass processing, or harsher industrial cabinet environments.

Compared with adjacent vision-driven manufacturing domains, where shared datasets and common benchmarks have accelerated progress, peen forming still lacks a unified evaluation foundation. This gap is therefore not only a data-availability issue, but also a benchmarking and standardization issue that slows cumulative progress, independent validation, and fair assessment of competing sensing and AI strategies. A structured roadmap for addressing this gap through open datasets and benchmark standardization is presented in Section 7.1.

The representative reported results further illustrate both the promise and the current limitation of the evidence base. In direct peen-forming studies, computer-vision-based coverage estimation has been reported at 92–95% on only 10–20 flat aluminum panels, while CNN-based coverage classification reaches 94% accuracy on 150 single-material 2024-T3 panels under clean laboratory conditions [38,59]. Within the broader reviewed evidence base for high-precision optical inspection, laser line scanner plus point-cloud processing has demonstrated approximately ±0.05 mm accuracy in clean laboratory settings, but with evidence still limited to about 50–200 total cases and with sensitivity to dust and scan-time constraints [103,105]. Closed-loop shot peen forming with in-process measurement and optimization has also been demonstrated; yet, current validation remains laboratory-scale and does not establish robust generalization across large curved aerospace panels or harsh cabinet environments [62]. Taken together, these examples confirm that experimental progress exists, but that the available evidence remains dominated by flat panels, single-material studies, clean-laboratory metrology, or small controlled demonstrations, with comparatively limited validation on complex curved aerospace panels and robust cabinet-scale closed-loop operation.

### 6.2. Reliance on Proxy Metrics with Weak Shape Link

Almen intensity and percent coverage continue to be used as the main tools for judging quality on the shop floor. The two are practical but indirect. Almen intensity is an internationally standardized indicator of near-surface compressive stress, but it does not provide a spatially resolved curvature map. It is sensitive to strip setup and modeling assumptions. At high coverage levels, Almen-based measurements may exhibit saturation effects, while the geometric evolution of the panel continues. Measurement techniques of residual stresses (XRD, hole drilling, neutron diffraction) each have depth limits, costs, or destructive natures. This complicates the generation of dense and spatially resolved labels, which supervised vision AI models require for shape prediction.

### 6.3. Limits of Current Process and Finite Element Models

Finite element (FE) models of peen or shot forming require extensive calibration to accurately capture full-panel deformation, particularly for large aerospace components. Discrete shot models, in comparison, are accurate but are expensive and require inputs that are hard to quantify, such as impact-velocity distributions and rate-sensitive plasticity. Eigenstrain or Almen-calibrated FE approaches can reduce the gap but have limitations of proxy metrics [78]. Consequently, such models are useful for generating artificial data and trend investigations, but are not yet capable of training AI models with ground truth, as can be performed on production parts in real settings.

### 6.4. Challenges of In-Process Vision in Peening Cabinets

Most vision systems that work well in welding or AM assume reasonably clear lines of sight. Peen cabinets do not offer that. Image quality and calibration are both adversely affected by airborne media, dust, specular metal skins, cabinet vibration, and windows. Digital image correlation (DIC) studies have reported sensitivity of optical measurement accuracy to dust, vibration, and reflective surfaces in industrial environments. Due to this, most teams measure ex situ (CMM, laser tracker, photogrammetry), followed by in-season updating of process plans. This kills the concept of in-process, closed-loop correction [185].

### 6.5. Minimal Demonstrations of Closed-Loop Control

There are a few promising research articles that demonstrate inverse peen forming planning and step-and-measure correction strategies [62]. However, they tend to be run on small scales and rely on ex situ metrology between successions. Also, they do not report long-term robustness or latency budgets. In many cases, industrial peen cabinets are not connected to external software to provide information on nozzle path, dwell time, or safety interlocks, and therefore, AI is simply running next to the machine and not in the control loop. Enabling vision-based AI recommendations to operate directly within real-time control loops, therefore, remains an unresolved practical challenge.

### 6.6. Difficulty of Generating Ground Truth

To validate vision-based generated curvature maps or curvature stress, we need a trusted reference. Several residual stress measurement techniques exist, including X-ray diffraction (XRD), hole drilling, and neutron diffraction; however, each presents limitations in penetration depth, spatial resolution, cost, or destructiveness. Hole drilling can disrupt the curvature field, and neutron diffraction, though accurate, is an expensive process with slow output. Hence, these methods are not useful for applying to hundreds or thousands of parts just to make ground truth to feed to AI models. Due to this challenging hazard, most case studies stopped at proxy targets (coverage, intensity, or relative deformation), and that is the reason supervised learning for peen forming limits its potential and remains data-starved.

### 6.7. Domain Shift Across Materials, Finishes, and Geometries

Although one model may perform well in one setup, it may exhibit significant performance degradation when the material or thickness of metal plates, size of shot, or even surface finishing is altered. The majority of investigations of vision-based coverage or deformation are conducted using clean aluminum plates with constant illumination, which cannot be directly applied to curved multi-reflective aerospace skins or darkened surfaces with shot residue. Even with the help of cold start or warm start using transfer learning concepts, there are still limited or almost no domain adaptation strategies and multi-site datasets until now.

### 6.8. Safety, Integration, and Lifecycle Constraints

In real peening cabinets, several constraints are imposed that are rarely mentioned by academic or laboratory-based prototypes. These include the visual line that must not be obstructed despite mechanical deflection. Calibration should remain stable even after mechanical shock, and purged window cleanliness must be maintained for safe optical view. There is very limited literature on the behavior of vision systems after weeks of operation, and whether illumination drifted or what recalibration frequency was achieved. These practical constraints contribute to industrial hesitancy regarding long-term robustness and maintainability of vision-based systems in peening environments. The overall AI techniques that can be adapted in peen forming require these practical considerations.

On the whole, even though literature demonstrates that vision-based AI for peen forming is technically viable, several things need to mature for this system to be called established. These include the following:

1. Widely adopted datasets and benchmarking frameworks are lacking;
2. Quality assessment remains predominantly based on indirect proxy metrics;
3. Physics-based models remain computationally intensive for large-scale deployment;
4. In-process optical sensing remains vulnerable to cabinet-specific environmental effects;
5. Demonstrations of fully autonomous closed-loop systems remain limited.

To make this realistic and industrially applicable, the following steps should be considered first.

### 6.9. Technology Readiness Level Assessment for Peen Forming

While vision-based AI systems have achieved high technology readiness levels (TRL 7–9) in welding, additive manufacturing, and CNC machining [31,33,175], peen forming implementations remain predominantly at research stages (TRL 2–4). Table 12, Table 13 and Table 14 provide a structured TRL assessment of specific technology combinations for peen-forming applications. To improve transparency and traceability, each TRL entry was interpreted using the operational evidence fields summarized in Table 11, rather than by maturity labels alone.

**Table 11:** Operational fields used for TRL assignment in this review.

Field	Categories Used in This Review	Role in Conservative TRL Assignment
Direct validation basis	Direct peen-forming validation; adjacent-domain transfer only; conceptual/no direct validation.	Transfer-only or conceptual evidence was not treated as equivalent to direct peen-forming demonstration.
Sample scale	<20, 20–49, 50–200, >200 panels/specimens/cases (or best available reported scale).	Larger and more diverse validation supported movement from early proof-of-concept toward representative validation.
Validation scenario	Simulation only; controlled laboratory; representative/pilot environment; near-industrial/production environment.	Environment fidelity constrained the upper plausible TRL bound.
Replication scope	Single study/site; multiple studies; independent replication across sites or conditions.	Replication increased confidence in maturity claims.
Uncertainty/reporting completeness	Explicit quantitative uncertainty or failure discussion; partial qualitative limitation reporting; no clear uncertainty discussion.	Incomplete reporting triggered conservative lower-bound interpretation.

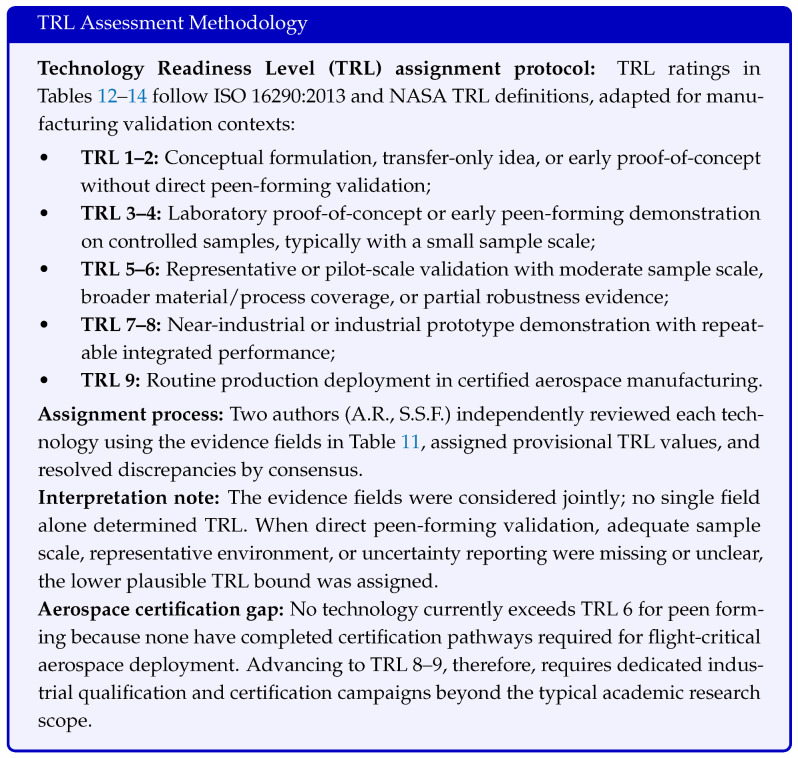



The quantitative evidence summarized in Table 12, Table 13 and Table 14 should be interpreted in this context. Several studies report encouraging results, including high coverage estimation accuracy in controlled imaging setups, sub-millimeter ex situ curvature measurement, and promising offline predictive performance. However, these results are still typically derived from limited sample counts, narrow material ranges, laboratory optics, or flat-panel validation. Evidence specifically addressing large, complex, and curved aerospace panels remains comparatively scarce, which is one of the main reasons why current peen-forming implementations remain at relatively low technology-readiness levels despite promising component-level results.

**Table 12:** Technology readiness assessment of vision–AI systems for peen forming (Part I: Coverage estimation and curvature measurement).

Technology Combination	Current TRL	Evidence Quality	Key Limitation/Bottleneck	Recommended Application	Path to Next TRL
**Coverage Estimation System**					
RGB Camera + Rule-Based Segmentation [59]	TRL 4	1 study (N = 10–20 flat panels) ^a^	Limited to low coverage (<150%); human-comparable accuracy (92–95%) but no curvature prediction; tested only on flat Al plates	Lab-scale coverage verification for research prototypes	Validate on curved panels; expand to Ti alloys; test in dusty cabinet conditions (TRL 5)
RGB Camera + CNN Classification [38]	TRL 4–5	1 study (N = 150 panels, single material) ^a^	Achieves 94% accuracy on clean 2024-T3; no public dataset; inference time 180 ms (too slow for real-time)	Offline quality inspection after peening; academic benchmarking	Optimize to <50 ms inference; create 200+ panel validation set from industrial cabinets (TRL 6)
RGB-D + U-Net Segmentation [35]	TRL 3–4	0 peen forming studies; extrapolated from sheet forming ^c^	Depth noise from specular reflection; no demonstrated peen forming application in the literature; concept proven in sheet forming	Coverage + shallow curvature mapping for non-aerospace applications	Acquire peening-specific training data (N > 100); deploy anti-reflective coatings; field test 50+ panels (TRL 5)
**Curvature Measurement System**					
Laser Line Scanner + Point Cloud Processing [103,105]	TRL 5–6	3 studies (N = 50–200 total) ^b^	±0.05 mm accuracy proven in clean lab; cabinet dust causes 20–30% data loss; requires 5–10 s full-panel scan	Ex situ final inspection with cleaned panels; small-batch aerospace production	Integrate compressed air purging; reduce scan time to <3 s via optimized toolpaths (TRL 7)
Structured Light + 3D Reconstruction [44,107,109]	TRL 4	0 peen forming studies; extrapolated from sheet forming ^c^	Pattern saturation on reflective Al/Ti; 5–15 s acquisition prohibits real-time; vibration sensitivity > 2 g	High-precision offline metrology for aerospace certification samples	Develop polarization filtering; fast single-shot patterns (<1 s); validate ± 0.02 mm on 100 panels (TRL 5–6)
Stereo Vision + Depth Estimation [113,116,117]	TRL 4	Concept demonstrated in related domains only ^c^	±0.5–2 mm accuracy insufficient for aerospace tolerances (±0.1–0.5 mm required); texture-dependent	Large automotive panels where ±1 mm tolerance acceptable	Integrate deep stereo matching (sub-pixel accuracy); hybrid with laser scanner for ground truth (TRL 5)
Photogrammetry (Multi-View SfM) [124,129,130]	TRL 5	2 validation studies (offline) ^d^	Sub-mm accuracy but 30–120 s processing time; not real-time; requires static multi-camera rig	Validation of FE models; large wing panel (>2 m) global geometry verification	Already mature for offline use; not suitable for closed-loop (remains TRL 5 for real-time)

^a^ Based on [38,59]; ^b^ Based on [103,105]; ^c^ No direct peen forming validation reported; ^d^ Based on [124,130]; Evidence-quality descriptors and conservative TRL assignment follow Table 4 and Table 11; Evidence quality assessment: Number of independent studies, sample size, validation environment (lab/pilot/production); where reporting was incomplete, lower-bound interpretation was used.

**Table 13:** Technology readiness assessment of vision–AI systems for peen forming (Part II: Predictive AI systems).

Technology Combination	Current TRL	Evidence Quality	Key Limitation/Bottleneck	Recommended Application	Path to Next TRL
**Predictive AI System**					
ANN/MLP for Almen → Arc Height [18,146]	TRL 5–6	5–8 studies (N = 50–150 each) ^e^	Proxy-based (no direct geometry); ±8–12% prediction error; validated on 50–150 samples	Process parameter optimization for existing Almen-based workflows	Widely used but fundamentally limited by Almen proxy; cannot advance without vision input
CNN + LSTM for Multi-Pass Curvature [51,62]	TRL 3–4	1 study (N=10–20 panels) ^f^	Demonstrated on 10–20 lab panels; 0.6 mm RMSE but requires a 150-panel training set (unavailable publicly)	Research tool for understanding curvature accumulation physics	Acquire 300+ multi-pass dataset with validated CMM ground truth; test generalization across materials (TRL 5)
Physics-Informed Neural Networks (PINNs) [55,56]	TRL 2–3	No peen forming implementation ^c^	Concept validated in sheet forming; no peen forming implementation; requires FE-model integration	Synthetic data generation to augment limited real datasets	Validate eigenstrain-PINN on 50 real panels; compare with FE predictions; quantify uncertainty (TRL 4)
GANs for Synthetic Data [145,165,186]	TRL 2–3	Concept only ^c^	Can generate realistic defect images but lacks physical consistency (curvature, stress)	Data augmentation for coverage/defect classifiers	Develop conditional GANs with physics constraints; validate generated data improves model accuracy by >10% (TRL 4)
Transformers for Long-Range Dependencies [52]	TRL 2	No peen forming application ^c^	No peen forming application; computationally expensive (>200 ms inference); overfits on small datasets	Future research for large (>1 m^2^) panel global deformation prediction	Requires 1000+ panel dataset; lightweight vision transformer variants; edge deployment feasibility (TRL 3–4)

^c^ No direct peen forming validation; ^e^ Based on [18,146] and related ANN/MLP studies; ^f^ Based on [62]; Evidence quality: Number of independent validations, sample sizes, and deployment environments; Evidence-quality descriptors and conservative TRL assignment follow Table 4 and Table 11; where reporting was incomplete, lower-bound interpretation was used.

**Table 14:** Technology readiness assessment of vision–AI systems for peen forming (Part III: Closed-loop control and edge deployment).

Technology Combination	Current TRL	Evidence Quality	Key Limitation/Bottleneck	Recommended Application	Path to Next TRL
**Closed-loop Control System**					
Vision + Manual Correction [25,62]	TRL 4–5	1 demonstration (N < 20) ^g^	Human-in-loop (operator adjusts parameters); 3–5 iteration cycles; tested on <20 panels total	Small-batch aerospace prototyping with expert oversight	Automate decision logic; validate on 100-panel production run with ≥90% first-pass success (TRL 6)
RGB-D + CNN + Rule-Based Control [31,98]	TRL 3	No integrated system ^c^	No demonstrated implementation; rule-based control too rigid for non-linear peening response	Proof-of-concept for constrained parameter space (single material, flat panels)	Implement on lab testbed; collect 200-panel closed-loop dataset with success metrics (TRL 4–5)
Multi-Sensor Fusion + MPC + OPC-UA [32,40,42]	TRL 3–4	Components demonstrated separately ^h^	All components proven separately (MPC in welding [31], OPC-UA in manufacturing [42]) but no integrated peen forming system	Future industrial adaptive peen forming with <200 ms loop time	Integrate testbed; validate latency budget; demonstrate 50-panel autonomous forming with ±0.5 mm tolerance (TRL 5–6)
Reinforcement Learning + Sim-to-Real [69]	TRL 2	No peen forming application ^c^	Proven in AM laser control but unstable with sparse peen forming rewards; safety concerns for aerospace	Long-term research direction for fully autonomous multi-objective optimization	Develop high-fidelity peen forming simulator; safe RL with human override; 500+ episode training (TRL 3–4)
**Edge Deployment Systems**					
Jetson AGX Xavier/Orin Inference [133,154]	TRL 5–6	Hardware validated; peen models not optimized ^i^	Hardware proven; peen forming models not optimized (pruning, quantization); latency 150–300 ms (target < 100 ms)	Real-time coverage/defect detection in research labs	Model compression (INT8, 50% sparsity); benchmark on peening-specific workloads; achieve < 80 ms (TRL 6–7)
FPGA Accelerators (Xilinx, Intel) [187]	TRL 4–5	Hardware validated in other domains ^c^	Ultra-low latency (<20 ms) but requires hardware expertise; no peen forming deployment reported	Safety-critical aerospace applications requiring deterministic timing	Port U-Net/ResNet to FPGA; validate 10,000-cycle reliability; integrate with industrial PLC (TRL 6)
Cloud-Edge Hybrid (5G) [188]	TRL 3	Concept only ^c^	5G latency (10–30 ms) acceptable but introduces cybersecurity risks for aerospace; unproven reliability	Non-critical applications with offline data analysis capability	Conduct latency/reliability field tests; address data sovereignty concerns; unlikely to reach TRL 7+ for aerospace

^c^ No direct peen forming validation; ^g^ Based on [62]; ^h^ MPC in [31], OPC-UA in [42]; ^i^ Based on [133,154]; Evidence quality: Integration maturity, component-level vs. system-level validation, deployment environments; Evidence-quality descriptors and conservative TRL assignment follow Table 4 and Table 11; where reporting was incomplete, lower-bound interpretation was used.

#### Critical Observations

The TRL assessment reveals three fundamental bottlenecks preventing industrial deployment:

1. Data scarcity: No technology exceeds TRL 5 due to lack of validated datasets (N > 200 panels). Even mature sensors (laser scanners at TRL 6–7 in other domains) remain TRL 5–6 for peen forming due to cabinet-specific challenges.
2. Integration gap: Individual components (sensors TRL 5–6, AI models TRL 3–5, control protocols TRL 6–7) have not been integrated into end-to-end systems. The highest TRL closed-loop demonstration is TRL 4–5 (manual correction) [62].
3. Aerospace certification barrier: Aerospace applications require TRL 8–9 (flight-proven), but current vision–AI systems lack the determinism, traceability, and failure-mode characterization necessary for compliance with aerospace manufacturing qualification and traceability requirements.

Advancing the field requires coordinated efforts: shared datasets (Section 7.1), standardized benchmarks, and multi-institutional validation campaigns. Progression toward aerospace-grade deployment will require coordinated efforts involving dataset standardization, validation campaigns, and integration with certified control architectures.

## 7. Forward-Looking Opportunities and Author Proposals

**Figure 6:**
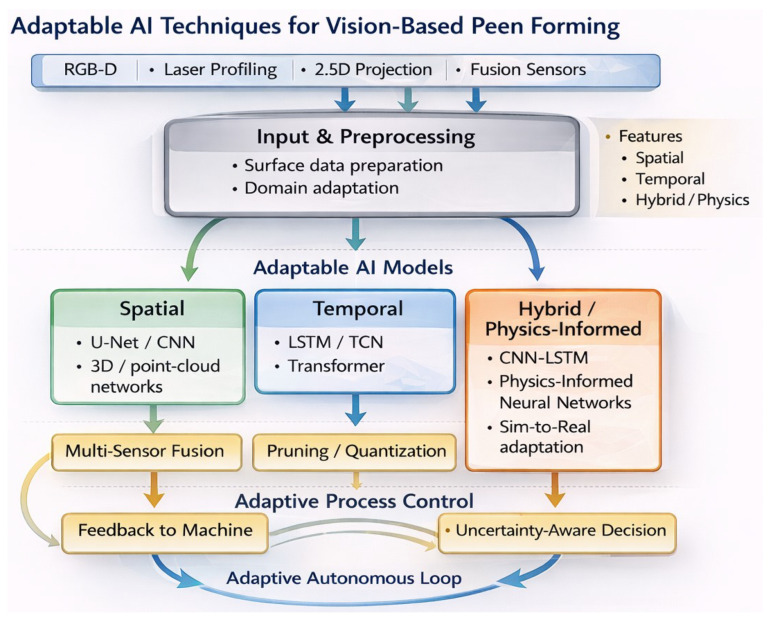
Adaptable artificial intelligence techniques and modeling strategies applicable to vision-based peen forming systems. The framework illustrates the processing pipeline from multi-modal visual inputs and preprocessing (e.g., RGB-D and laser fusion, 2.5D projection) to spatial learning models such as U-Net, convolutional neural networks (CNNs), and three-dimensional point-cloud networks. Temporal modeling approaches, including long short-term memory (LSTM), temporal convolutional networks (TCNs), and transformer architectures, are shown for capturing deformation evolution across peening passes. Hybrid and fusion models, including CNN–LSTM combinations, generative adversarial networks (GANs) for domain adaptation, and physics-informed neural networks (PINNs), enable improved generalization and physical consistency. Deployment-oriented components, such as model pruning and quantization, uncertainty-aware physics integration, edge and real-time control, reinforcement learning, and machine feedback, support closed-loop and adaptive peen-forming operation.

In the previous section, we identified the main barriers to deployment, including the lack of shared datasets, laboratory-constrained validation, reliance on proxy metrics, and the limited demonstration of closed-loop systems. These gaps also indicate the most practical directions for progress. The present section is intentionally forward-looking: unless explicitly stated otherwise, the material below should be interpreted as author synthesis and conceptual proposal, rather than as direct empirical evidence from the screened review corpus. Where evidence from adjacent manufacturing domains is used to motivate these proposals, it is treated as transferable contextual support rather than as direct peen-forming validation. In this context, the current maturity levels and advancement pathways of sensing, prediction, closed-loop control, and edge deployment are summarized in Table 12, Table 13 and Table 14, while the adaptable end-to-end AI pipeline is illustrated in Figure 6.

### 7.1. Conceptual Proposal: Open Datasets and Peen-Forming Benchmarks

To address the dataset and benchmarking gap identified in Section 6.1, the field needs a shared benchmark framework rather than isolated laboratory datasets. A useful benchmark should cover representative materials and panel geometries, synchronized vision data acquired under both controlled and cabinet-constrained conditions, clearly documented process parameters, and validated geometric ground truth. Core benchmark tasks should include coverage estimation, curvature reconstruction, deviation mapping, and defect-region detection, with multi-pass prediction added where temporally resolved data are available.

A staged release strategy is more realistic than prescribing a single fully mature industrial dataset at the outset. Early releases can focus on well-characterized laboratory panels with reliable post-process metrology, while later phases can extend to larger curved components, harsher optical conditions, and cross-cabinet variability. The key requirement is therefore standardization of data content and evaluation protocols rather than one fixed dataset format. Until common benchmarks emerge, comparison across sensing modalities, learning strategies, and deployment claims will remain difficult to reproduce and difficult to validate fairly.

### 7.2. Sim-to-Real and Synthetic Data

Collecting representative cabinet data is expensive and time-consuming, whereas FE- and eigenstrain-based simulations can generate large amounts of deformation and stress data. Although simulated outputs do not naturally include sensor noise, reflectivity artifacts, or cabinet disturbances, domain-adaptation strategies such as noise injection, style transfer, and adversarial alignment can narrow the sim-to-real gap [189]. A digital-twin framework combining the peening process, material behavior, and sensing layout could therefore provide a practical route for scaling supervised training while reducing dependence on costly experimental campaigns.

### 7.3. Multi-Sensor Fusion as the Default, Not the Exception

Single-modality vision systems remain vulnerable in peening cabinets because occlusion, airborne media, specular reflection, vibration, and window contamination can degrade performance. A more robust direction is therefore multi-sensor fusion, for example, combining RGB-D for wide-area coverage with laser scanning for local precision and, where useful, infrared or fixture-based signals. Fusion may be implemented through simple channel-level combination or more advanced architectures such as transformer-based or graph-based models. Standardized reporting of calibration, synchronization, and fusion design is essential for reproducibility and cross-laboratory generalization.

### 7.4. Edge AI and Real-Time Loops

Most current studies adopt an ex situ workflow (process → remove panel → scan → analyze), which limits real-time corrective capability. That is useful for research, but not for industry. If we want to make corrections after each pass or cycle, inference has to happen inside the cabinet controller or at least very close to it. It means compressed models (pruned CNNs, 2D–2.5D inputs, quantized weights) running on an IPC, Jetson, or FPGA, not a cloud GPU. Once we can reliably output results saying “these zones are under-formed” in under 200 ms, we can connect that to simple rule-based correction, e.g., re-pass this stripe, increase dwell here. Even partial inter-pass correction would represent a substantial advancement beyond the predominantly offline validation approaches reported to date.

### 7.5. Physics-Informed and Uncertainty-Aware AI

Industrial deployment requires not only predictive accuracy but also traceability, robustness, and uncertainty quantification. Physics-informed neural networks (PINNs) and constraint-augmented loss formulations incorporating curvature smoothness, elastic–plastic bounds, and geometric continuity constraints (smooth curvature, upper limits on deflection for given intensity, and geometric continuity near stiffeners) can keep neural networks from predicting garbage with noisy inputs. A reliable and trustworthy system is required. One solution is to include uncertainty estimation in case the system does not perform well, and mention that the results are not confirmed; please measure again. This is because some areas in peen forming are always hard to access, like edges and occluded ribs, so mistakes can be a possible outcome of the model. This way, at least they can be reassured that the model is performing well.

### 7.6. Conceptual Proposal: Closed-Loop Adaptive Peen Forming Architecture

The ultimate goal of vision-based AI in peen forming is closed-loop adaptive control, but no integrated peen-forming system has yet been demonstrated at industrially validated maturity. A realistic near-term pathway is therefore to define the architecture only at a high level: inter-pass sensing captures updated surface state, lightweight preprocessing converts measurements into stable geometric representations, and an AI inference layer estimates deviation maps or correction-relevant indicators on local edge hardware. These outputs can then be passed to a supervised decision layer that recommends bounded parameter updates or localized re-peening, while the cabinet controller and human–machine interface retain safety interlocks, operator override, and full traceability.

Evidence from welding, additive manufacturing, sheet forming, and CNC machining indicates that low-latency vision–AI feedback is technically plausible, but peen forming still lacks cabinet-validated demonstrations at comparable maturity [31,32,33,35,43,175,176]. For that reason, the most credible path remains staged: human-in-the-loop diagnostic assistance first, bounded corrective recommendations second, and only then constrained automation once robustness, uncertainty handling, and process traceability have been demonstrated across representative materials, geometries, and cabinets.

### 7.7. Cross-Domain Transfer (Use What Is Already Working)

Cross-domain transfer remains one of the most practical acceleration routes for peen forming. Welding, additive manufacturing, sheet forming, and CNC machining have already demonstrated useful solutions for harsh-optics sensing, temporal modeling, and multi-sensor integration, and these components can be adapted to peen forming with substantially less effort than fully de novo development. Transfer of optical filtering, sequential prediction, and fusion strategies from these domains may also reduce the amount of peen-forming data required for initial model development.

### 7.8. Industrialization and Lifecycle

Industrial adoption will depend not only on model accuracy but also on calibration discipline, traceability, maintenance, drift monitoring, and operator usability. In practice, deployment requires standard operating procedures for sensor cleaning and recalibration, version-controlled model updates, fail-safe fallback modes, and clear operator-facing interfaces that translate model outputs into actionable zone-based guidance. These lifecycle considerations are essential if vision–AI tools are to move from promising laboratory prototypes to auditable production assets.

## 8. Conclusions

Peen forming remains a strategically important dieless process for shaping large, thin metallic panels; yet, its monitoring and control workflows are still dominated by indirect proxies and retrospective inspection. This review shows that vision-based AI offers a credible route toward more observable and adaptive peen forming by combining modern optical sensing with spatial, temporal, and physics-aware learning models. Across the surveyed literature, the most promising sensing options are laser-based and RGB-D systems, potentially complemented by multi-sensor fusion where cabinet conditions justify the added complexity. Related manufacturing domains further demonstrate that deformation mapping, predictive modeling, and low-latency feedback are technically achievable in harsh environments, providing transferable design patterns for peen forming.

At the same time, industrial deployment remains constrained by limited validated datasets, continued reliance on proxy metrics, harsh cabinet optics, costly ground-truth acquisition, and the scarcity of cabinet-validated closed-loop demonstrations. Progress, therefore, depends less on new conceptual architectures than on shared benchmarks, stronger experimental validation on representative geometries, robust edge deployment, and conservative integration strategies that preserve traceability and operator oversight. With these foundations in place, peen forming can evolve from an empirically tuned operation into a measurable, auditable, and progressively adaptive manufacturing process.

## Figures and Tables

**Table 1:** Terminology definitions and usage conventions.

Term	Definition and Usage in This Review
**Process Terminology**	
Peen forming	A dieless metal forming process that uses controlled shot impacts to induce curvature through residual stress gradients. Primary focus of this review.
Shot peening	Surface treatment process primarily for fatigue life improvement; shape modification is incidental. Used when citing literature that does not explicitly address forming.
Laser peen forming	Variant using laser-induced shock waves instead of mechanical shot; excluded from scope unless vision–AI methods are directly transferable.
**Process Parameters**	
Almen intensity	Standardized measure of peening energy (SAE J442), expressed as arc height in units of “A” (e.g., 0.012 A = 0.012 inches = 0.305 mm).
Coverage	Percentage of surface area impacted by shot. Entire surface impacted at least once at 100%; 200% = twice the minimum exposure time for full coverage.
Shot size	Diameter of spherical media (mm), typically 0.4–0.8 mm (S110–S230 per AMS 2431).
Shot velocity	Impact speed (m/s), typically 40–80 m/s for pneumatic systems.
**Geometric Quantities**	
Curvature	Refers to principal curvatures $\kappa_{1},\kappa_{2}$ (1/radius) unless otherwise specified. Gaussian curvature ($\kappa_{1}\kappa_{2}$) and mean curvature (($\kappa_{1}+\kappa_{2})/2$) are specified where relevant.
Springback	Elastic recovery after shot impact removal, typically 5–15% of total deformation for aluminum alloys [16].
Tolerance	Permissible deviation from target geometry. Aerospace: ±0.1–0.5 mm for wing skins [17]; automotive: ±0.5–1.5 mm.
**AI and Computing**	
Real-time	In this review: inference latency < 200 ms, enabling inter-pass corrections during 5–10 s dwell periods. Not hard real-time.
Edge AI	Model inference on local hardware (Jetson, FPGA, industrial PC) rather than cloud servers.
TRL	Technology Readiness Level (ISO 16290:2013 scale 1–9) adapted for manufacturing.
Ground truth	Validated reference measurements (CMM ±0.01 mm, laser tracker ±0.05 mm, XRD ±30 MPa).
**Vision Sensing**	
RGB-D	Combined color (RGB) and depth imaging via structured light or time-of-flight.
Depth map	Two-dimensional array of distance measurements (mm).
Point cloud	Set of 3D coordinates (x,y,z).
Occlusion	Regions where sensor line-of-sight is blocked.
Specular reflection	Mirror-like reflection from polished surfaces saturating sensors.

**Table 2:** Representative vision-and-AI studies relevant to peen forming.

Research Direction	Vision Technology	AI Technique	Application Domain	Ref.
Imaging systems and techniques for fusion-based metal additive manufacturing; case studies on in situ (during process)/ex situ (after process) imaging and artificial intelligence integration	High-speed cameras, thermal cameras, digital cameras, pyrometer-based thermal cameras; stereo vision, structured-light three-dimensional imaging, interferometry	Transfer learning, data augmentation; artificial intelligence/machine learning for defect detection, process control, quality optimization	Additive manufacturing (metal), laboratory prototype, not peen forming	[60]
Computer vision methods for estimating coverage in peen-formed aluminum plates	No mention found; image segmentation (implied two-dimensional imaging)	Inductive rule-based segmentation, multi-agent segmentation system	Peen forming (aluminum plates), research prototype, low coverage only	[59]
Review of artificial intelligence in additive, subtractive, and hybrid manufacturing; focus on process monitoring and control	No mention found; vision-based techniques for in situ monitoring	Convolutional neural networks, support vector machines, transfer learning; machine learning for defect detection, process control, surface roughness prediction	Additive, subtractive, hybrid manufacturing; industrial context, not peen forming	[61]
Systematic review of artificial intelligence for control in laser-based additive manufacturing	No mention found	Reinforcement learning; artificial intelligence for process control and monitoring	Laser-based additive manufacturing; industrial pilot/full deployment, not peen forming	[69]
Literature review of image-based quality inspection in smart manufacturing systems	Digital camera systems (type not specified)	Deep neural networks, deep learning	Smart manufacturing systems; industrial pilot/full deployment, not peen forming	[70]

**Table 3:** Structured summary of the literature search and screening workflow.

Workflow Stage	Procedure Used in This Review	Outcome/Exclusion Basis
Database search	Searches were conducted in Scopus, Web of Science, and IEEE Xplore within the formal screened-corpus window from 1 January 2025 to 31 July 2025.	Records were exported with available metadata for structured review screening.
De-duplication	Duplicate records were removed using combinations of title, DOI, and venue information.	A de-duplicated candidate set was prepared for relevance filtering and screening.
Vision/sensing relevance filtering	Records were filtered for relevance to vision sensing, AI, monitoring, or control.	Studies outside the review scope were removed before detailed screening.
Title/abstract screening	Two reviewers screened titles and abstracts using the predefined inclusion and exclusion criteria.	Studies lacking sufficient relevance to vision-based AI for peen forming or transferable manufacturing contexts were excluded.
Full-text eligibility assessment	Full texts were evaluated through the same dual-review process.	Principal exclusion categories included lack of vision/AI monitoring relevance, insufficient methodological or validation detail, inaccessible/non-English full text, and overlapping publication versions.
Final screened corpus	Included studies comprised direct peen-forming papers and representative adjacent-manufacturing studies used for transferability and maturity analysis.	These studies formed the qualitative synthesis base for the comparative review.
Contextual/foundational references and later contextual updates	Background references, standards, historical sources, and limited later contextual additions were tracked separately.	These references were not treated as part of the formal screened review corpus.

*Note:* Exact stage-wise numeric counts were not retained in a verifiable screening log; therefore, the workflow is reported here transparently in procedural form rather than with reconstructed counts.

## Data Availability

This review is based on a systematic analysis of peer-reviewed literature. All cited references are publicly available through institutional library access or open-access repositories. No new experimental data were generated for this study. Any benchmark specification discussed in the manuscript is presented as a conceptual research direction rather than an existing public dataset.
